# The comparative mortality of an elite group in the long run of history: an observational analysis of politicians from 11 countries

**DOI:** 10.1007/s10654-022-00885-2

**Published:** 2022-06-23

**Authors:** Philip M. Clarke, An Tran-Duy, Laurence S. J. Roope, Jay A. Stiles, Adrian G. Barnett

**Affiliations:** 1 Health Economics Research Centre, Nuffield Department of Population Health, University of Oxford, Oxford, OX3 7LF UK; 2 Centre for Health Policy, Melbourne School of Population and Global Health, University of Melbourne, Melbourne, Australia; 3 Australian Centre for Health Services Innovation and Centre for Healthcare Transformation, School of Public Health and Social Work, Queensland University of Technology, Brisbane, QLD Australia

**Keywords:** Inequality, Mortality, Life-expectancy, Politicians

## Abstract

This study aims to compare the mortality rate and life expectancy of politicians with those of the age and gender-matched general populations. This was an observational analysis of mortality rates of politicians (i.e. members of national parliaments with available data on dates of birth, death and election, gender, and life tables) in 11 developed countries. Politicians were followed from date of first election until either death or the last available year with life table data. Relative mortality differences were estimated using standardised mortality ratios (SMRs). Absolute inequalities were quantified as the difference in survival by deducting a population’s remaining life expectancy from politicians’ remaining life expectancy at age 45, estimated using Gompertz parametric proportional hazards models. We included 57,561 politicians (with follow-up ranging from 1816–2016 for France to 1949–2017 for Germany). In almost all countries politicians had similar rates of mortality to the general population in the early part of the twentieth century. Relative mortality and survival differences (favouring politicians) increased considerably over the course of the twentieth century, with recent SMRs ranging from 0.45 (95%CI 0.41–0.50) in Italy to 0.82 (95%CI 0.69–0.95) in New Zealand. The peak life expectancy gaps ranged from 4.4 (95% CI, 3.5–5.4) years in the Netherlands to 7.8 (95% CI, 7.2–8.4) years in the US. Our results show large relative and absolute inequalities favouring politicians in every country. In some countries, such as the US, relative inequalities are at the greatest level in over 150 years.

**Supplementary Information:**

The online version contains supplementary material available at 10.1007/s10654-022-00885-2.

## Introduction

There has been considerable interest in using historical data to investigate how inequalities have evolved over long periods of time [1–3]. The recent focus has been long-run trends in ‘top incomes’ which represent the share of income accruing to the richest in society. A finding common to many countries has been that top income shares fell dramatically in the first half of the twentieth century, but have been rising since the 1980s [2]. There is less evidence on the long-term evolution of inequalities within society in other aspects of welfare, such as life expectancy. A few studies have provided evidence on the life expectancy differences between countries and regions [4]. These studies showed that in many high-income countries, the large gains in life expectancy experienced for many decades have recently stalled, and within-country regional inequalities in life expectancy have widened [4].

There is less research on within-country differences in life expectancy between socio-economic groups [5, p.113], in part because the mortality information has only recently begun to be routinely linked with other socio-economic data in many countries. A recent review suggests that health inequalities within societies have widened over the last few decades [6]. For example, in the US the life expectancy differential for those in the top 5% compared with the bottom 5% increased by up to 3 years between 2001 and 2014 [7].

What is the evidence on life expectancy gaps prior to this? While evidence is somewhat fragmented and conflicting, the prevailing view has somewhat been shaped by Antonovsky’s early influential review [8],which provides examples of socio-economic differentials in mortality dating back to the early part of the nineteenth century. This is consistent with evidence from the 1860s of greater mortality among non-taxpayers than taxpayers in the US [9], and of mortality gaps by socioeconomic status in France [10]. However, this view is not universally held and emerging evidence from Sweden, based on long-term registers covering mortality differentials between occupations since the early nineteenth century, show gradients in adult mortality emerging in Sweden only in the second half of the twentieth century [11, 12]. Based on historical data from seven studies in Europe, the US and Canada, another study questions any causal link between income and mortality, and argues that associations observed between income and mortality today are probably a recent phenomenon [13]. Deaton in his book *The great escape: health, wealth, and the origins of inequality* has also emphasized that the differentials we currently observe were not always present [14]. An often-cited study comparing average life expectancy at birth of British noble families and the general population between 1541 and 1871 showed a divergence in life expectancy only after 1750 [15]. Unfortunately it is hard to extend this approach as many countries do not have aristocracies and the status of these elite groups has changed dramatically over time in many societies in terms of both power and wealth [16–18].

Politicians are another elite group. In contrast to nobility, historical birth and death data are available in many countries. It is therefore possible to compare long-run trends in politicians’ mortality with those of the populations they represent for many more countries than in previous comparisons involving royals or nobility. To date, examinations of comparative mortality of politicians have been confined to just a few countries (e.g. those in the UK [19] and the Netherlands [20]). Our objective was to conduct a wider examination of long-run relative and absolute inequalities. Our findings are intended to complement work on long-term health inequalities as well as the trajectories of ‘top incomes’ [1–3] and thereby gain a broader understanding of the evolution of inequality within and between countries.

## Materials and methods

### Data sources and verification

For the general population’s mortality data we used life tables from both the Human Mortality Database (HMD) [21] and the Human Life Table (HLT) Database [22]. This was an observational analysis of mortality rates of politicians. We collated existing data from 11 countries (Australia, Austria, Canada, France, Germany, Italy, the Netherlands, New Zealand, Switzerland, the UK, and the US) that had good biographical information on politicians (in either chamber of national parliaments).

The variables required for our analyses were politicians’ gender, dates of birth and death, and dates they attained office. As the dates of death are not always recorded, we undertook extensive checking against other sources such as Wikidata and Wikipedia. We also checked the complete details for a randomly selected subset of politicians (10 per country), and for any politicians who had apparently died within a few months of taking office, entered politics before age 21, or were apparently still alive beyond 102 years.

The tracking of politicians’ deaths is often passive in the sense that it relies on news reports of their deaths, rather than on active contact during the follow-up period. If contact is lost with some politicians, their deaths will not be recorded which would bias the standardised mortality ratios (SMRs) downward. To examine the degree to which this unknown loss to follow-up may affect the SMRs, a robustness check was undertaken by re-estimating the SMRs for different lengths of follow-up time (as censoring at a fixed follow-up time reduces both the propensity and impact of unknown loss to follow-up). We started with a follow-up time of 10 years, meaning that we only counted deaths within the first 10 years post-election and censored those politicians still alive. We repeated this for follow-up periods of 10–60 years.

### Statistical analysis

All analyses were performed using R software version 3.1.0 or higher and public data on politicians and life tables is available for most countries (see supplementary materials for details). The study was approved by Melbourne University Ethics ID 1,853,298.1.

#### Relative mortality

For each country we began in the first calendar year with data both from life tables and on politicians’ election dates. We followed each politician from election date (i.e. time at risk) until either death or the last available year with life table data. Matching to election date is a way to avoid the potential for immortal time bias, which may arise as politicians will tend to die at an older age than the general population simply because they must be elected to office to become a politician, and cannot die prior to that [23]. We estimated relative differences in mortality using standardised mortality ratios (SMRs) [24]. This involved: (1) matching each politician by country, year at risk, age at risk and gender to the relevant period life table, which captures the mortality experience of a population at a period in time [25]; and (2) calculating the SMR as the observed number of deaths in each year divided by the number expected based on mortality rates from life tables.

In the year that politicians first assumed office, the expected number of deaths were adjusted on a pro rata basis according to the proportion of the year remaining following taking office. For example, if a politician was elected halfway through a year, their exposure time was halved.

Some early life tables in six of the countries were produced every 5 or 10 years and so we interpolated the gaps in the tables to produce annual life tables. We used a two-dimensional spline for age and year. We visually checked the plot fit by comparing the model predictions with available years and checked the residuals for skewness and outliers.

To summarize overall trends in relative inequality, we plotted the SMRs against calendar years and used a spline to estimate the trend and its associated 95% credible interval [26–28]. The spline was estimated using Poisson regression models with the count of deaths as a dependent variable and the expected number of deaths as an offset. The trend was modelled using a b-spline basis with two or three degrees of freedom, and also a linear trend [27]. The best fitting model for the trend was chosen as that with the smallest deviance information criterion, which is a trade-off between model fit and complexity [28].

Over time, the cohorts of politicians became older relative to the general population, making their age structure different from that of the general population. To control for the potential impact of changes in age structure, we conducted sensitivity analyses in which we also employed direct standardization [29] using weights from the World Standard Population [30]. We also stratified by gender by rerunning all analyses on male and female subgroups.

#### Absolute inequality

To quantify changes in absolute inequalities between politicians and the general population, we estimated gaps in their respective remaining life expectancies from the age of 45 years (mean age of entry into parliament). For politicians, we fitted Gompertz parametric proportional hazards models [31] using age as time scale to data in consecutive 10-year time windows. These models were used to estimate the remaining life expectancy of a politician in each time window, which was then compared with the remaining life expectancy of the general population in the same time window. The proportion of male politicians was far larger than that of female politicians, and life expectancy of males is generally shorter than that of females. We therefore used proportions of male and female politicians in 10-year time windows as weights to calculate the pooled life expectancies of the corresponding general populations for the comparisons. To estimate the 95% CI for each remaining life expectancy, we bootstrapped the dataset 1,000 times and re-estimated the remaining life expectancy using the method described above.

## Results

**Fig. 1:**
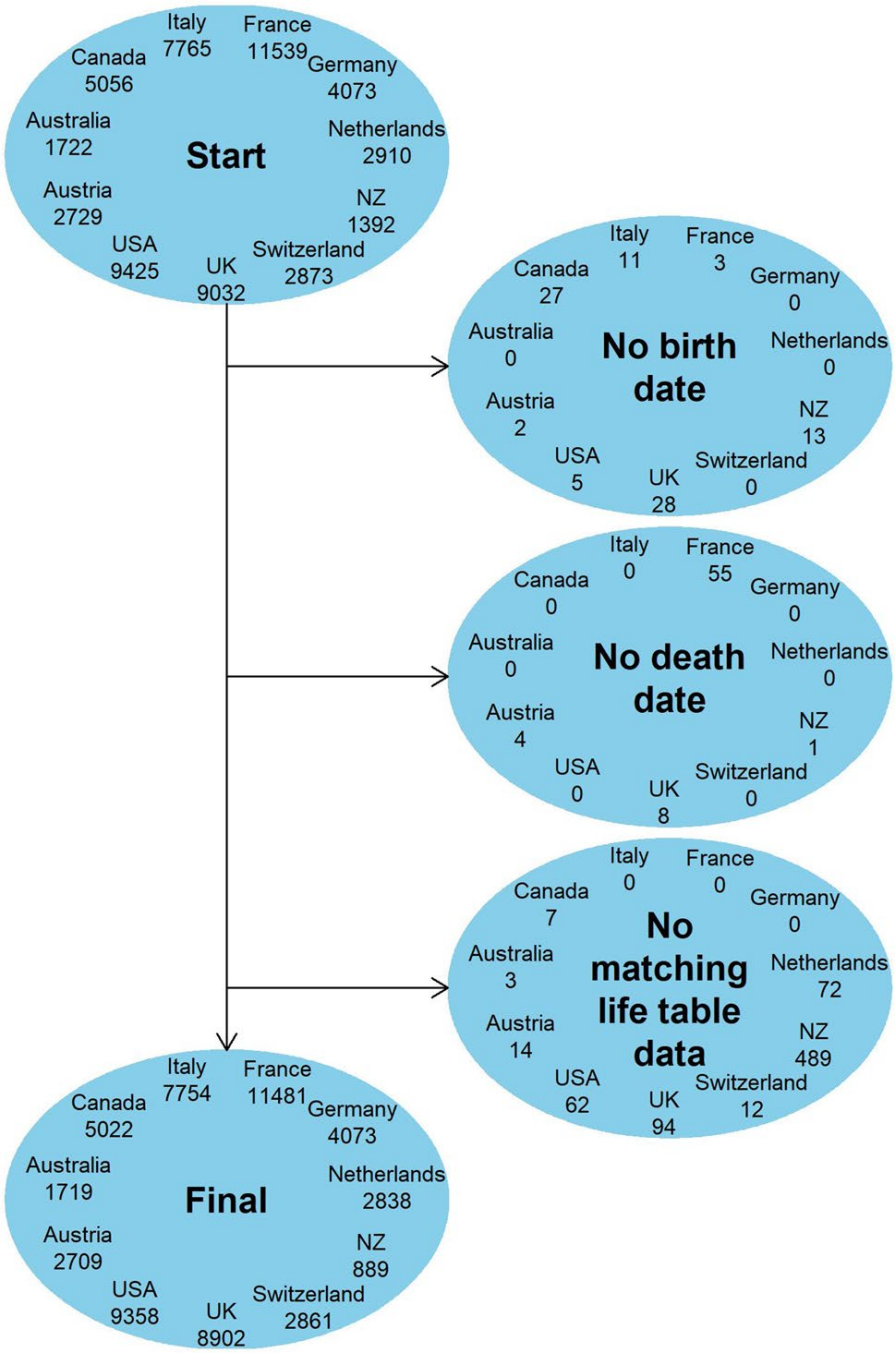
Flow diagram for selection of the final dataset and reasons for exclusions. Note: Politicians were excluded if date of birth or death were missing, or if there were no matching life-table data

Data were available from the nineteenth century for seven of the 11 countries: Germany, Canada, Netherlands, New Zealand, Switzerland, UK and the US. The level of missing data due to a lack of birth or death dates was low (< 1%) (Fig. 1). Only in one country (New Zealand) did a lack of historical life table data result in a large reduction of the sample size. Across all countries there were 57,561 politicians, with 40,637 deaths, giving a combined 2.6 million years of follow-up with a mean of 46 years per politician. Table 1 reports key descriptive statistics. The follow-up period ranged from 1816–2016 for France to 1949–2017 for Germany and the mean age at election was between 43 to 47 years, with female politicians constituting between 3 to 21% of the sample.

**Table 1:** Descriptive statistics for available politicians by country

Country	N	% female	N dead	Mean age first elected	Mean years of follow up	Year first elected	Last year followed up
Australia	1719	12	970	44	44.9	1901	2016
Austria	2664	16	1563	46	46.7	1918	2017
Canada	5022	8	3530	47	47.3	1867	2016
France	11,481	3	9935	45	46.0	1816	2016
Germany	4073	21	1767	45	46.1	1949	2017
Italy	7754	11	3381	47	47.9	1945	2014
Netherlands	2838	16	1811	46	46.5	1850	2016
New Zealand	889	14	513	44	44.7	1891	2014
Switzerland	2861	9	2013	47	47.7	1876	2016
UK	8902	5	7373	43	43.2	1838	2016
US	9358	3	7781	46	46.3	1850	2016

### Relative mortality

**Fig. 2:**
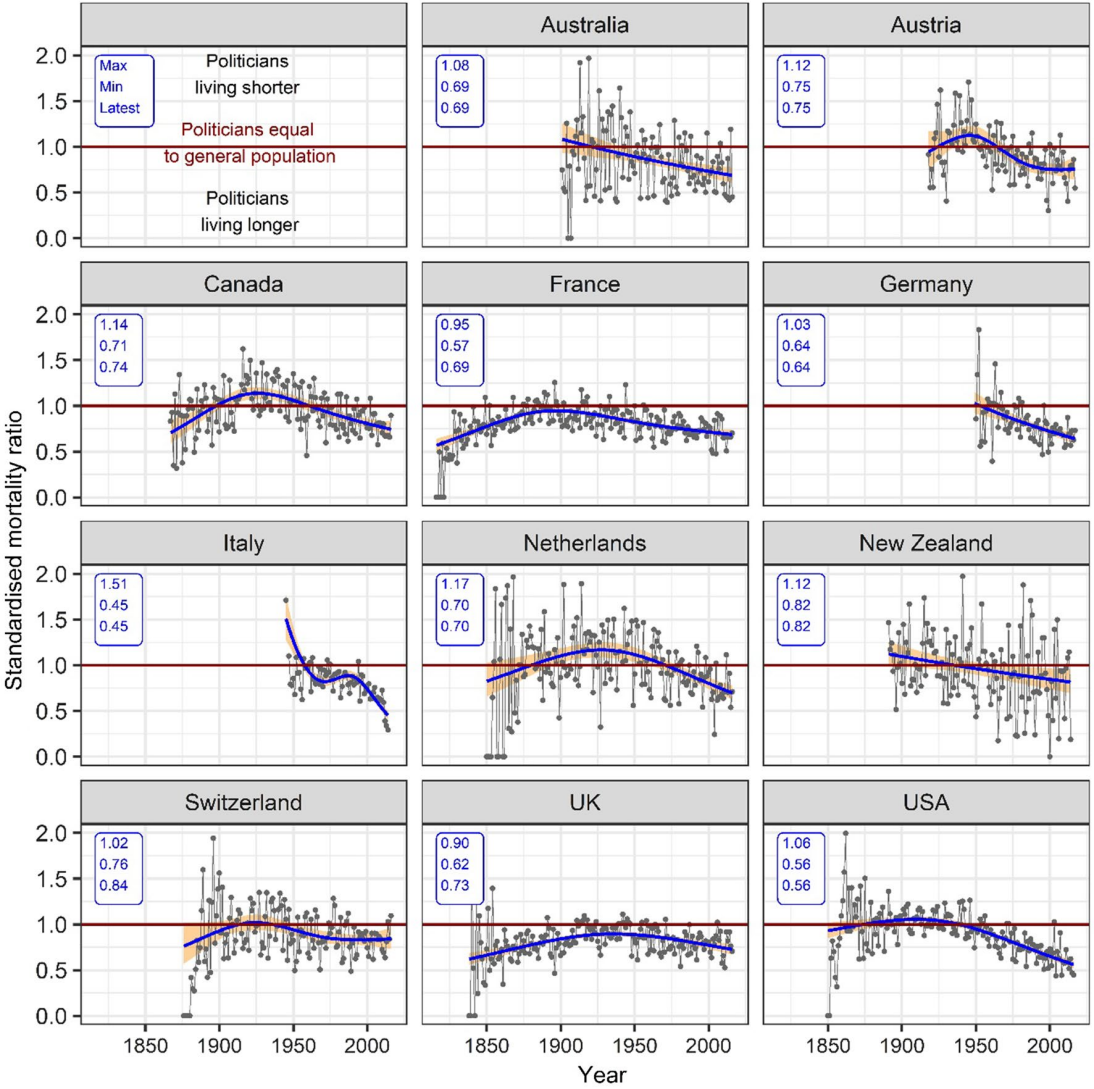
Standardised mortality ratios of politicians compared with the general population in 11 countries. Notes: Solid blue lines are the trend in standardised mortality ratio estimates. Shaded orange areas are the 95% CIs

Figure 2 shows a consistent pattern of changes in SMRs across countries. In all countries with data for the nineteenth century, the SMRs increased during that period, from below one to values approximately equal to one (except for the UK where SMRs increased but remained below one). This indicates that mortality rates of politicians generally became closer to those of the general population towards the end of the nineteenth century. In two of these countries (Canada and the Netherlands) politicians had a higher mortality rate than the general population in the late 19th and early twentieth century. Over the course of the twentieth century, SMRs declined, reflecting an increasing survival advantage for politicians relative to the general populations. However, there was considerable variation between countries in the extent of the survival advantage. In the most recent years the SMRs (95% CI) ranged from 0.84 (0.73 to 0.96) and 0.82 (0.69 to 0.95) in Switzerland and New Zealand, to 0.56 (0.52 to 0.61) and 0.45 (0.41 to 0.50) in the US and Italy.

The results using SMRs adjusted for changes in age structure were very similar to the unadjusted results but with larger CIs (see supplementary materials, Fig. S4). An animation (see supplementary materials, Fig. S5b) displaying the change in SMRs as a result of changes in follow-up periods showed that the smoothed SMR was smaller than one in every country and for all years, and increased with increasing follow-up time.

### Absolute inequality

**Fig. 3:**
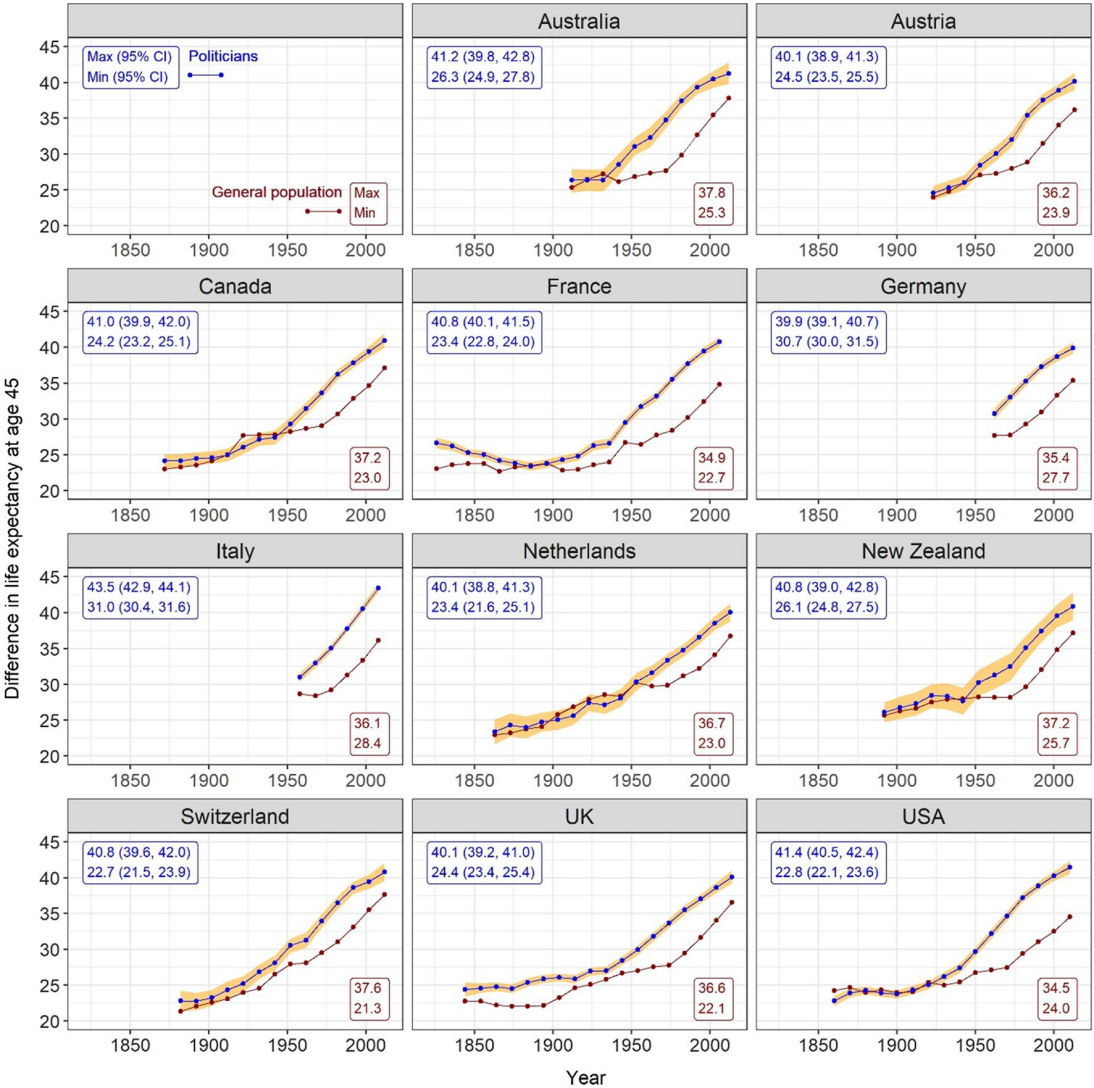
Remaining life expectancies at age 45 for politicians and general populations in 11 countries. Note. Shaded orange areas are the 95% CIs of politicians’ life expectancies

The remaining life expectancies at age 45 of both politicians and the general population have increased steadily over time since the early twentieth century (Fig. 3). In recent years (2011–2017), the life expectancies of politicians aged 45 years were remarkably similar between countries, ranging from 39.9 (95% CI 39.1–40.7) years in Germany to 43.5 (95% CI 42.9–44.1) years in Italy. During the nineteenth century, in those countries with data, the life expectancies of politicians slightly increased or remained largely unchanged in all countries except for France, where they slightly decreased.

**Fig. 4:**
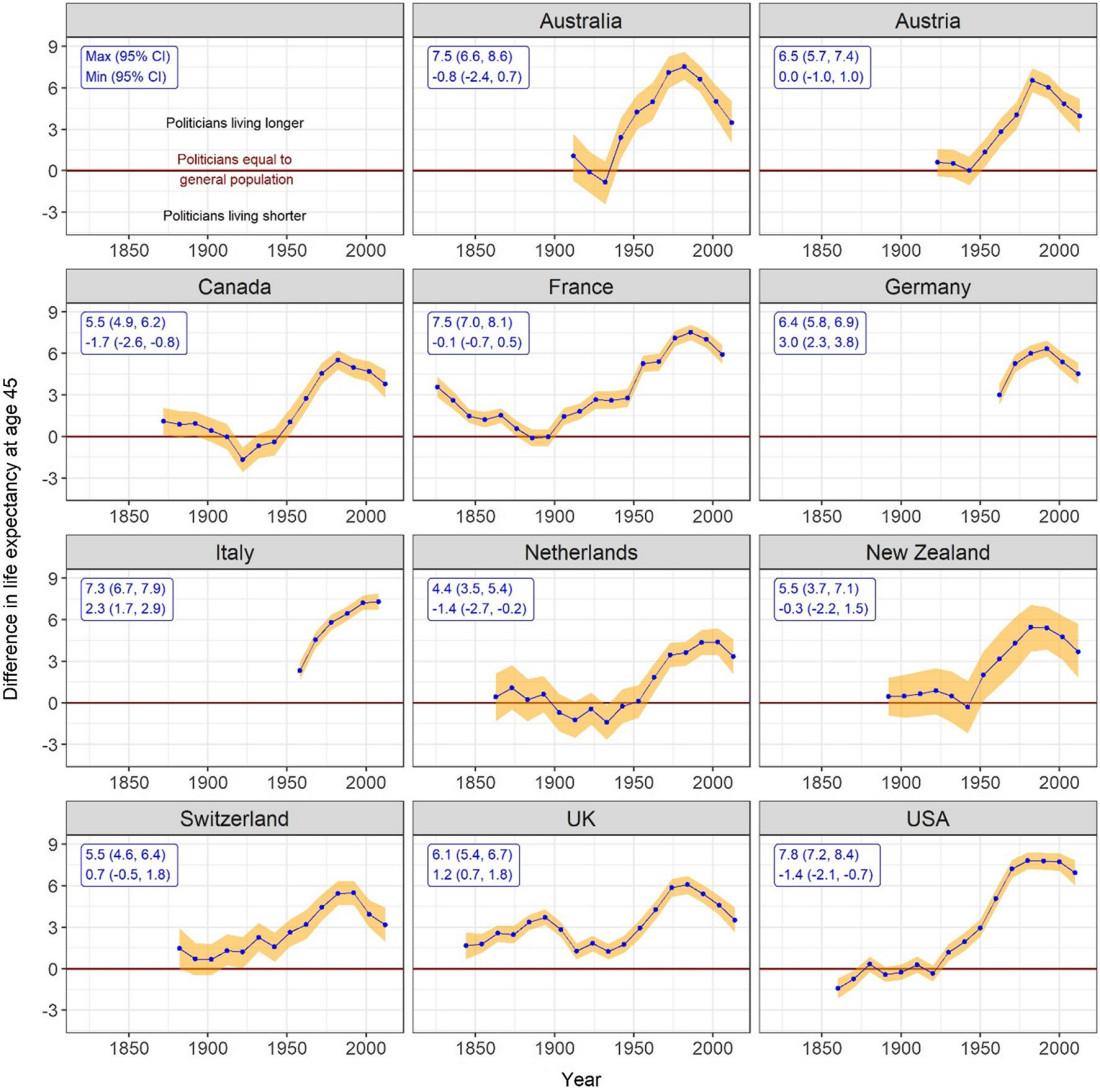
Gaps in the remaining life expectancies at age 45 years between politicians and general populations in 11 countries. Notes: Positive (negative) values of difference in remaining life expectancy aged 45 indicate that politicians have a greater (lower) remaining life expectancy aged 45 than the general populations. Shaded orange areas are the 95% CIs

The differences in life expectancies between politicians and the general population were consistent across all countries (Fig. 4). Life expectancy gaps increased during the second half of the twentieth century, with maximum life expectancy gap ranging from 4.4 (95% CI 3.5–5.4) years in the Netherlands to 7.8 (95% CI 7.2–8.4) years in the US. While the life expectancy gaps for some countries have recently decreased (showing a different trend to recent movements in relative inequalities in mortality), the gaps are still much greater than those prior to 1950. Results by gender (which are broadly similar to those reported in the main text) are reported in supplementary Figs S6–S10).

## Discussion

We have demonstrated how health inequalities can be measured retrospectively over long time periods by comparing the mortality of politicians with that of the populations they represent. There were some notable and consistent patterns across all countries. We found that current politicians have lower mortality levels, in both relative and absolute terms, than the general populations. The mortality differentials between politicians and the general populations widened considerably over the second half of the twentieth century in all countries. The smallest differences in mortality between politicians and the general populations were in the late 19th and early twentieth century. In several countries, the most recent SMRs are similar to those observed in the middle of the nineteenth century, indicating that relative mortality differences are at their greatest level in the last 150 years.

What can account for these observed trends? A potential framework for considering this question is Mackenbach’s recent essay, which argues that long-term trends in population health are governed by the rise and fall of successive waves of diseases [32]. If these waves of disease are more severe in certain sub-populations, this will affect observed trends in health inequalities.

Take tobacco use, which increased over the first half of the twentieth century before declining as evidence on its dangers emerged [33] While information on health behaviours of politicians is limited, it is interesting to note that 8 of the 10 US Presidents who have been identified as smokers died between 1850 and 1950 [34]. This includes several who died of smoking related diseases, such as President Ulysses Grant who died of throat cancer [35]. High rates of smoking in the first half of the twentieth century were not confined to politicians. Aggregate consumption of tobacco products grew rapidly following the introduction of cigarettes [36], and smoking was prevalent across society, including high status socio-economic groups. For example, a landmark survey of British doctors in 1951 classified only 13% as non-smokers [37], and unlike today the prevalence of smoking in this professional group was the same as the general British population at that time [38]. Large epidemiological studies estimated that smoking contributed up to a decade of lower life expectancy [39]. So, if rates of smoking declined faster among politicians than the general public, this may in part explain the emergence of life expectancy gaps in many countries after 1950.

Another potential explanation for the increase in politicians’ survival advantage in the second half of the twentieth century is the expanded range of therapies to treat cardiovascular disease. For example, President Franklin Roosevelt suffered hypertension (with his blood pressure recorded as 230/126 mm Hg in 1944), as did Prime Minister Winston Churchill (recorded as 160/90 mm Hg in 1949) [40]. Both of these political leaders died of stroke. At that time, there were debates about whether hypertension was harmful [41]. Long-term epidemiological studies after the Second World War, notably the Framingham study, helped resolve this debate as they indicated that hypertension could reduce life expectancy by around 5 years [42]. The subsequent emergence of therapies to prevent cardiovascular disease, starting with antihypertensive drugs diuretics in 1959 [43], have likely improved human health over the last 60 years [44]. If take-up of these medications was faster among politicians than the general public, this may help to explain the increasing gaps. Such a pattern is consistent with one of the recent Swedish studies covered in our introduction [11], which showed that higher status occupations actually were at a greater risk of death from circulatory diseases prior to the 1960s, but since that time have been at significantly lower risk.

Finally, what is the role of infectious diseases? For diseases such as COVID-19 that involve person-to-person contact, politicians are potentially at a greater risk, as they are likely to experience high rates of population mixing, particularly during election campaigns. A French study [45] has examined this issue using data from government elections in March 2020 at the outbreak of the COVID-19 pandemic, but did not find excess mortality in politicians. Beyond transmission, the other key factor that may influence mortality is standard of care, and there may be differentials in the standard of care between politicians and the public. For example, in countries such as the United States politicians were some of the first to receive COVID-19 vaccines [46] and the former US President received treatment that was estimated to have cost more than half a million US dollars when he contracted COVID-19 [47].

Does holding political office directly impact mortality? The findings are mixed. A recent analysis of close elections in the US indicates that winners live longer than losers by around a year [48]. In contrast, another study involving heads of state from 17 countries found that winners had a slight survival disadvantage compared with runners-up [49]. It is also possible that a selection mechanism (e.g. advent of television broadcasting) changed the type of person who became a politician and this may impact on observed trends. This could be explored in future work using additional co-variates on politicians that are available in some countries (e.g. university education).

Today, the survival advantage of politicians remains very high compared with that observed in the first half of the twentieth century. It has, however, recently declined slightly in absolute terms in some countries. This has been driven by the life expectancy of the general populations recently increasing at a faster rate than that of politicians. Whether this trend continues will depend in part on future trends in overall life expectancy. Triggered by the COVID-19 pandemic, life expectancy declined from 2019 to 2020 [50]. While this very recent trend might reverse, there was evidence from prior to the pandemic in some high-income countries, particularly in the US and UK, indicating declining gains to life expectancy of the general population that gives grounds for concern [51–54].

Politicians’ salaries in many countries are well above the average population levels [55]. For example, incomes of politicians in Australia were between two and six times the average wage over the twentieth century [56]. In most countries, in the first half of the twentieth century, there was a dramatic fall in inequality, as measured by the share of overall income and wealth accruing to the richest in society (‘top income shares’) [2]. This fall in top income/wealth shares generally continued during the immediate post-war decades, before starting to rise in the 1980s [2]. The SMRs in our study paint a rather different picture. Though they too indicate a fall in inequality in the early twentieth century, relative mortality gaps began rising much earlier (before 1940 in all 11 countries) than did top income shares.

Our study has some limitations. As there were no female politicians before 1920 in most countries, the cohorts of politicians attaining their offices before 1920 were compared only with males from the general populations. There was unknown loss to follow-up due to passive tracking of politicians’ deaths. While this might lead to biased estimates of the health inequalities, our sensitivity analysis showed that changes in the follow-up time did not change the trends in survival advantage of the politicians.

We conducted our study for high-income, democratic countries and therefore the evolution of health inequalities we found might not be generalizable to low and middle-income countries. However, the methods and approach in this study can be more widely applied to understand the long-run evolution of health inequalities in potentially any country. It would also be possible to employ similar methods for other elite groups, such as judges, if the necessary data are available. For example, an analysis of judges on the US Supreme Court found their mortality to be similar to that of the general population until 1950, but lower thereafter [57]. Building a greater evidence across more countries and other elites provides a long-run view that can help inform policy debates on how to close gaps in life-expectancy between elites and average citizens.

## Supplementary Information

Below is the link to the electronic supplementary material.

Supplementary file1 (DOCX 5322 kb)
