# Supplementary material for: The comparative mortality of an elite group in the long run of history: an observational analysis of politicians from 11 countries
Source: Eur J Epidemiol. 2022 Jun 23;37(9):891–9. doi: 10.1007/s10654-022-00885-2 (PMC9223251; doi:10.1007/s10654-022-00885-2)

# The comparative mortality of an elite group in the long

# run of history: an observational analysis of politicians from 11 countries

# Supplementary Materials

# S1 Sources of data

# Table S1a: Life Table data

|  | Human Mortality database  (https://www.mortality.org/) | | Additional data sources (Unless otherwise stated from the Human Life Table (HLD) database) |
| --- | --- | --- | --- |
| Country | Start Year | End Year |  |
| [Australia](https://www.mortality.org/cgi-bin/hmd/country.php?cntr=AUS&level=1) | 1921 | 2014 | Early data (from 1900 to 1920) from http://www.abs.gov.au/AUSSTATS/abs@.nsf/DetailsPage/3105.0.65.0012008?OpenDocument |
| [Austria](https://www.mortality.org/cgi-bin/hmd/country.php?cntr=AUT&level=1) | 1947 | 2017 | HLD data for Austria:  (years: 1865-1882, 1889-1892, 1899-1912, 1930-1933) |
| [Canada](https://www.mortality.org/cgi-bin/hmd/country.php?cntr=CAN&level=1) | 1921 | 2011 | HLD data for Canada  (years: 1831, 1841, 1851, 1861, 1871, 1881, 1891, 1901, 1911, 1921) |
| [France](https://www.mortality.org/cgi-bin/hmd/country.php?cntr=FRA&level=2) | 1816 | 2016 | NA |
| [Germany](https://www.mortality.org/cgi-bin/hmd/country.php?cntr=DEU&level=2) | 1956 | 2013 | HLD data for Germany  (years: 1946-1947, 1949-2012) |
| [Italy](https://www.mortality.org/cgi-bin/hmd/country.php?cntr=ITA&level=1) | 1872 | 2014 | NA |
| [Netherlands](https://www.mortality.org/cgi-bin/hmd/country.php?cntr=NLD&level=1) | 1850 | 2016 | NA |
| [New Zealand](https://www.mortality.org/cgi-bin/hmd/country.php?cntr=NZL&level=2) | 1948 | 2013 | HLD data for New Zealand in (years: 1891-1915, 1934-1938) |
| [Switzerland](https://www.mortality.org/cgi-bin/hmd/country.php?cntr=CHE&level=1) | 1876 | 2016 | NA |
| [United Kingdom](https://www.mortality.org/cgi-bin/hmd/country.php?cntr=GBR&level=2) | 1922 | 2016 | HLD data for United Kingdom (years: 1838-1854, 1861-1963) |
| [United States](https://www.mortality.org/cgi-bin/hmd/country.php?cntr=USA&level=1) | 1933 | 2016 | Michael R. Haines Estimated Life Tables for the United States, 1850-1900  HISTORICAL WORKING PAPER 0059; DOI 10.3386/h0059  ISSUE DATE September 1994 & All HLD data for United States of America (years: 1900-1932) |

# Table S1b: Data on politicians by country

| Country | Source |
| --- | --- |
| [Australia](https://www.mortality.org/cgi-bin/hmd/country.php?cntr=AUS&level=1) | The Australian Parliamentary Handbook contains historical information on the Australian Parliament - Members since 1901  https://parlinfo.aph.gov.au/parlInfo/download/handbook/newhandbook/2008-12-19/toc_pdf_repeat/Part%206%20-%20Historical%20information%20on%20the%20Australian%20Parliament.pdf;fileType=application%2Fpdf  We obtained an electronic version from the Australian Parliamentary Library (parliamentary.librarian@aph.gov.au) which covers both Senators & Members of the House of Representatives. |
| [Austria](https://www.mortality.org/cgi-bin/hmd/country.php?cntr=AUT&level=1) | Data available at a public source: [https://www.parlament.gv.at/WWER/PARL/](https://protect-au.mimecast.com/s/u4V6C1WZKqhzVA07SY5XP7?domain=parlament.gv.at)  An electronic version which covers the “Provisional and Constituent National Assembly, National Council, Federal Council” was obtained from Parliament Directorate. |
| [Canada](https://www.mortality.org/cgi-bin/hmd/country.php?cntr=CAN&level=1) | Data available at a public source: https://lop.parl.ca/sites/ParlInfo/default/en_CA/People |
| [France](https://www.mortality.org/cgi-bin/hmd/country.php?cntr=FRA&level=2) | Data available at public source: [http://www2.assemblee-nationale.fr/sycomore/resultats](https://protect-au.mimecast.com/s/zDM4C2xZLrcv0PAqSprVB8?domain=www2.assemblee-nationale.fr)  Covers the Assemblée Nationale. |
| [Germany](https://www.mortality.org/cgi-bin/hmd/country.php?cntr=DEU&level=2) | Obtained from GESIS - Leibniz-Institut für Sozialwissenschaften Abt. Datenarchiv für Sozialwissenschaften (DAS)- Data Archive for the Social Sciences (DAS) from database of parliamentarians i.e. member of the German Bundestag compiled by Prof. Wilhelm H. Schroeder. |
| [Italy](https://www.mortality.org/cgi-bin/hmd/country.php?cntr=ITA&level=1) | Obtained from Senate Library, Reference Service, Piazza della Minerva, 38 00186 Roma Italy.  Covers the Italian Senate and Chamber of Deputies. |
| [Netherlands](https://www.mortality.org/cgi-bin/hmd/country.php?cntr=NLD&level=1) | Data was purchased from PDC Informatie Architectuur BV. Covers both of members of Parliament and Senate |
| [New Zealand](https://www.mortality.org/cgi-bin/hmd/country.php?cntr=NZL&level=2) | Roll of Members of the New Zealand House of Representatives, provided by Parliamentary Service Te Ratonga Whare Pāremata, Private Bag 18041, Wellington 6160, New Zealand |
| [Switzerland](https://www.mortality.org/cgi-bin/hmd/country.php?cntr=CHE&level=1) | Data can be downloaded Council Members since 1848 can be downloaded from: https://www.parlament.ch/en/ratsmitglieder |
| [United Kingdom](https://www.mortality.org/cgi-bin/hmd/country.php?cntr=GBR&level=2) | Data obtained from an electronic database of MPs back to 1832 http://www.historyofparliamentonline.org/research/members |
| [United States](https://www.mortality.org/cgi-bin/hmd/country.php?cntr=USA&level=1) | Based on a combination of data from Biographical Directory of the U.S. Congress, and the chronological list of U.S. Senators: <https://bioguide.congress.gov/> and  <https://voteview.com/data>; |

# S2. Supplementary R code to create the standardised mortality ratios and plot the trends over time.

# <https://github.com/agbarnett/pollies/tree/master/R>

**S3. Supplementary R code to estimate life expectancies and plot the trends over time.**

# <https://github.com/antranduy/pollies/tree/master/R>

**S4. Sensitivity analysis results: standardised mortality ratios adjusted for age structure**

Fig. S4. Standardised mortality ratios adjusted for age structure

#
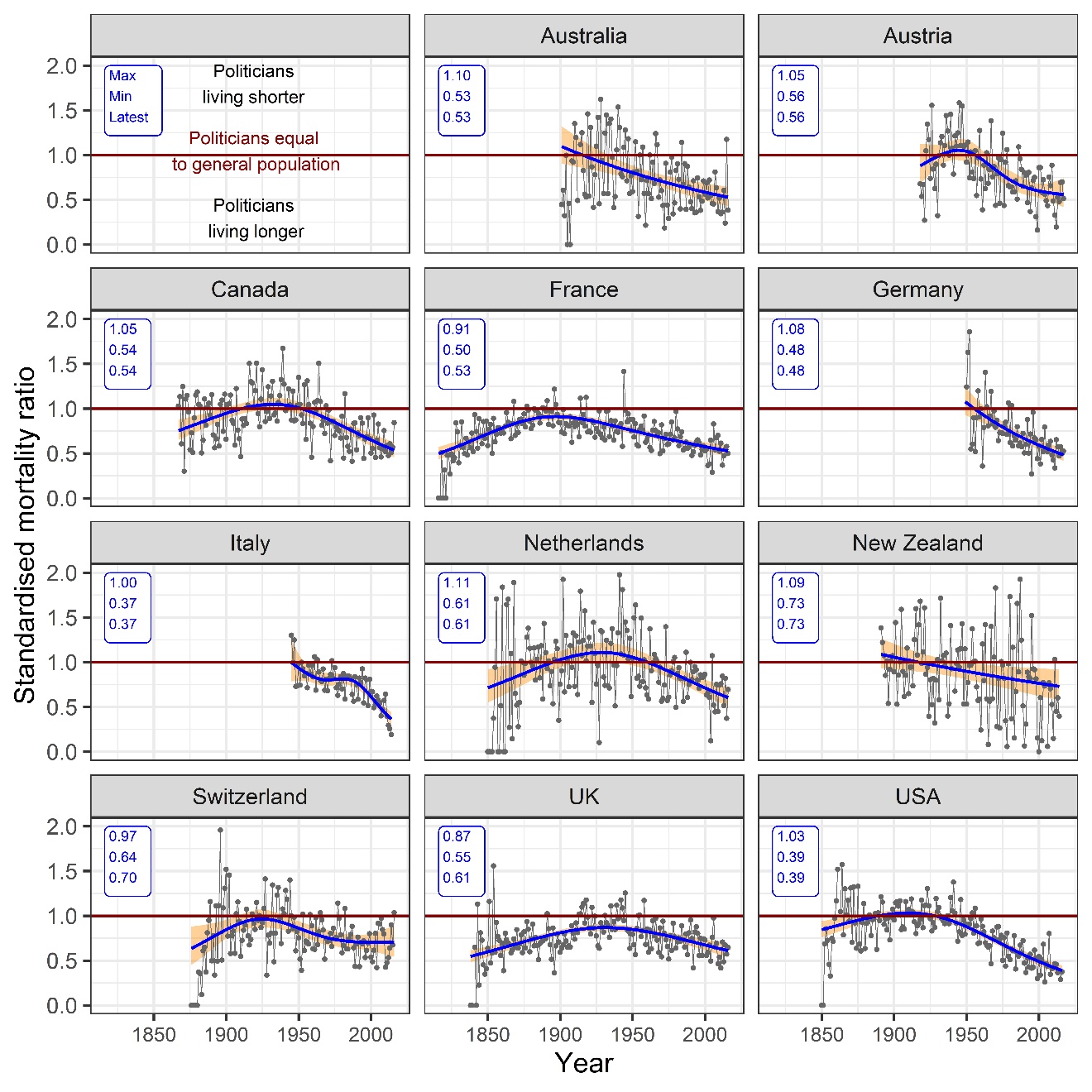


# Standardised mortality ratios in this figure were adjusted to make comparisons over time based on a constant

# age structure in each country. Solid blue lines indicate the trend in standardised mortality ratio estimates.

Shaded orange areas indicate the 95% CIs. These results were very similar to the unadjusted results but with larger CIs. The larger CIs are not surprising, as much more weight was given to younger ages, where there were fewer deaths and so more uncertainty.

# S5. Sensitivity analysis results: Standardised mortality ratios associated with different Lengths of the period of follow-up

To examine the degree to which this unknown loss to follow-up may affect the SMR estimates, a robustness check was made by re-estimating the SMRs for different lengths of follow-up time (as censoring at a fixed follow-up time reduces both the propensity and impact of unknown loss to follow-up). We started with a follow-up time of 10 years, meaning that we only counted deaths within the first 10 years post-election and censored those politicians still alive. We repeated this for follow-up for 10 to 60 years (see Fig S5a,b).


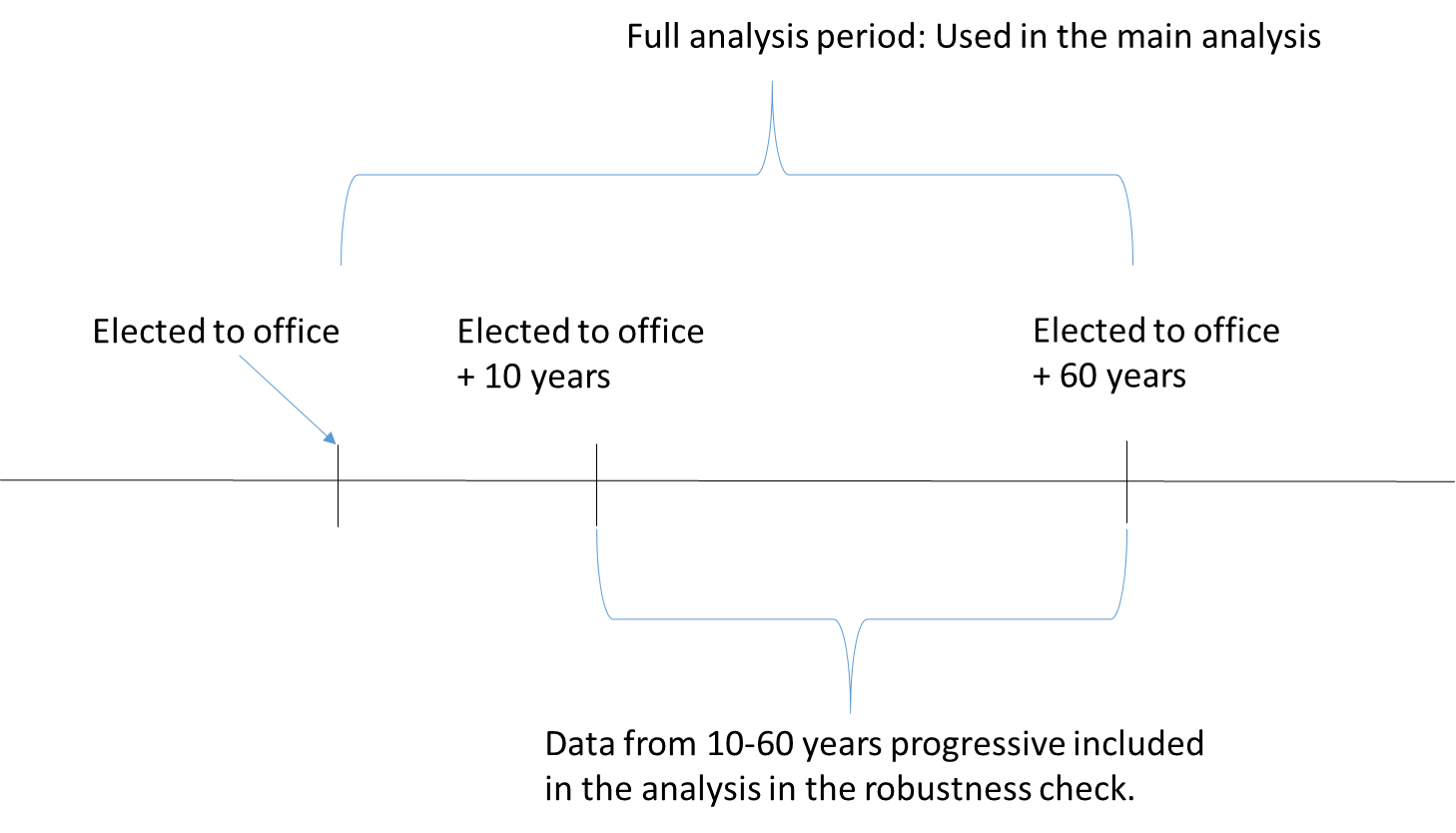


Fig S5a. Follow-up periods in the robustness check.

Below are some screenshots for Australia of analysis undertaken using four follow-up times.

**
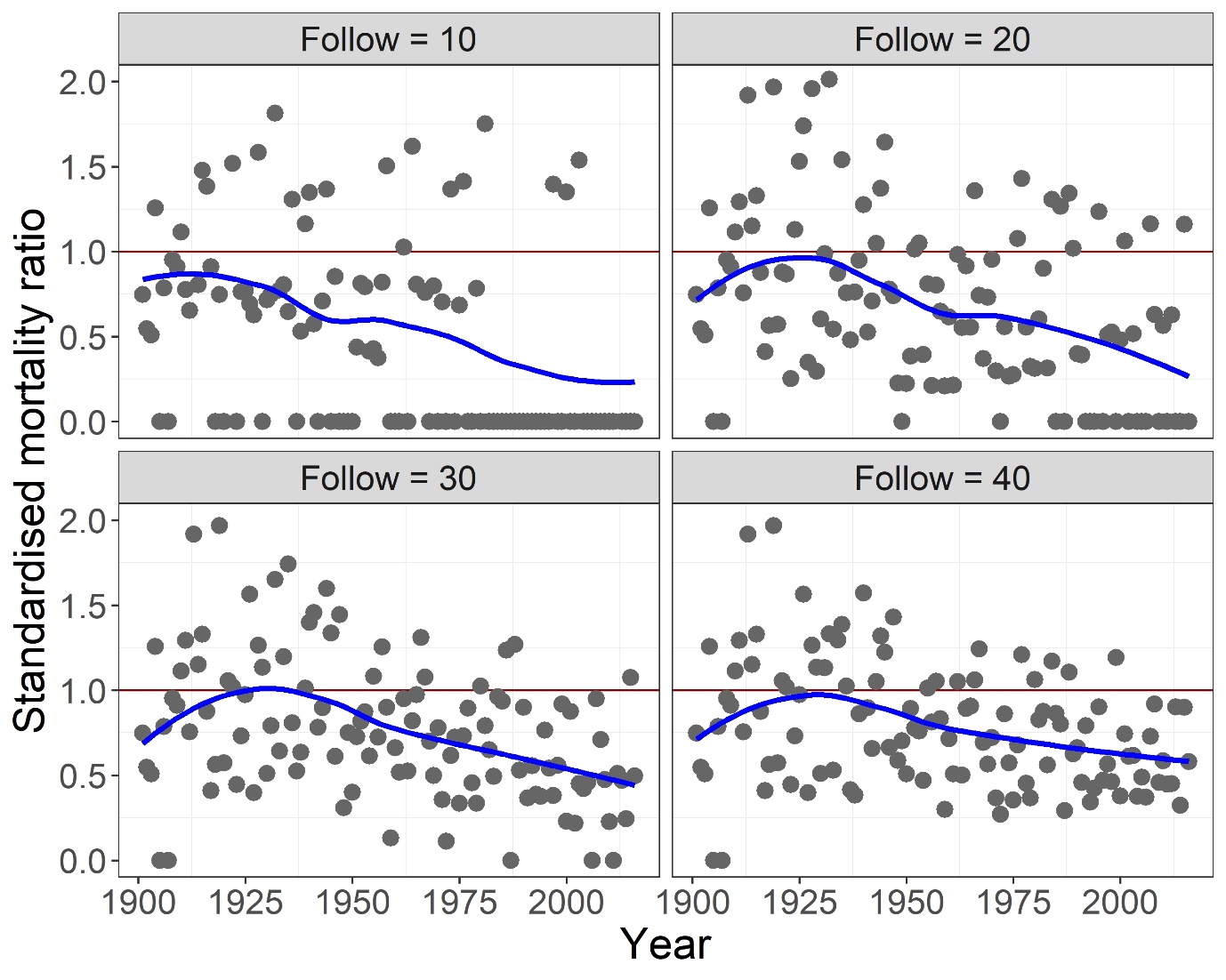
**

The rest can are contained in animations that are available on Github.

Fig S5b. Animation of the changes in standardised mortality ratios as a result from changes in lengths of period of follow-up. See GIF images at <https://github.com/agbarnett/pollies/tree/master/animations>

**S6. Sub-group analyses: standardised mortality ratios for female politicians**

Fig. S6. Standardised mortality ratios over time for female politicians

**
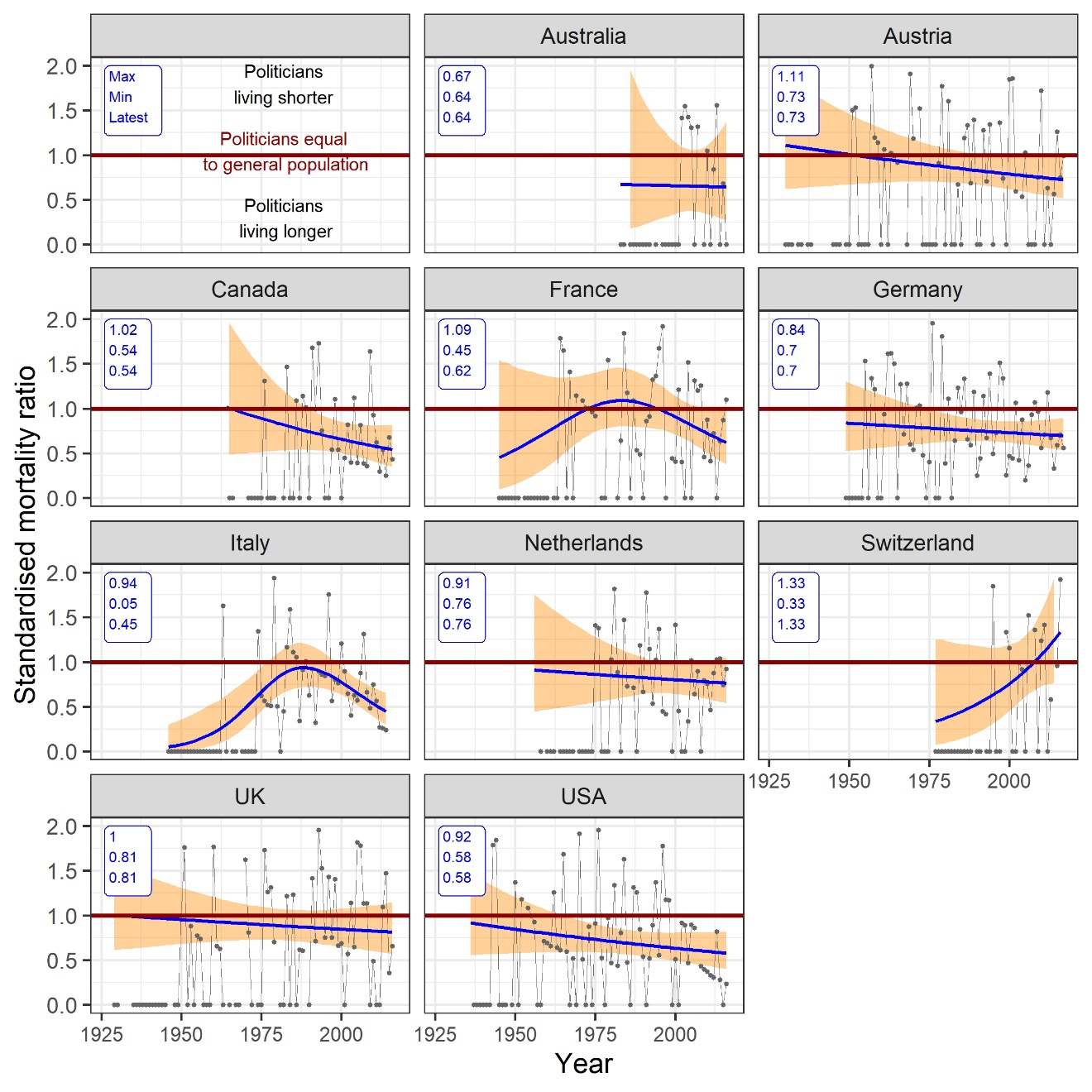
**

# NOTE: There are no results for New Zealand because there were too few female politicians. Solid blue lines indicate the trend in standardised mortality ratio estimates. Shaded orange areas indicate the 95% CIs.

**S7. Subgroup analysis: Remaining life expectancies for males**

Fig. S7. Remaining life expectancies at age 45 for male politicians and general populations in 11 countries.


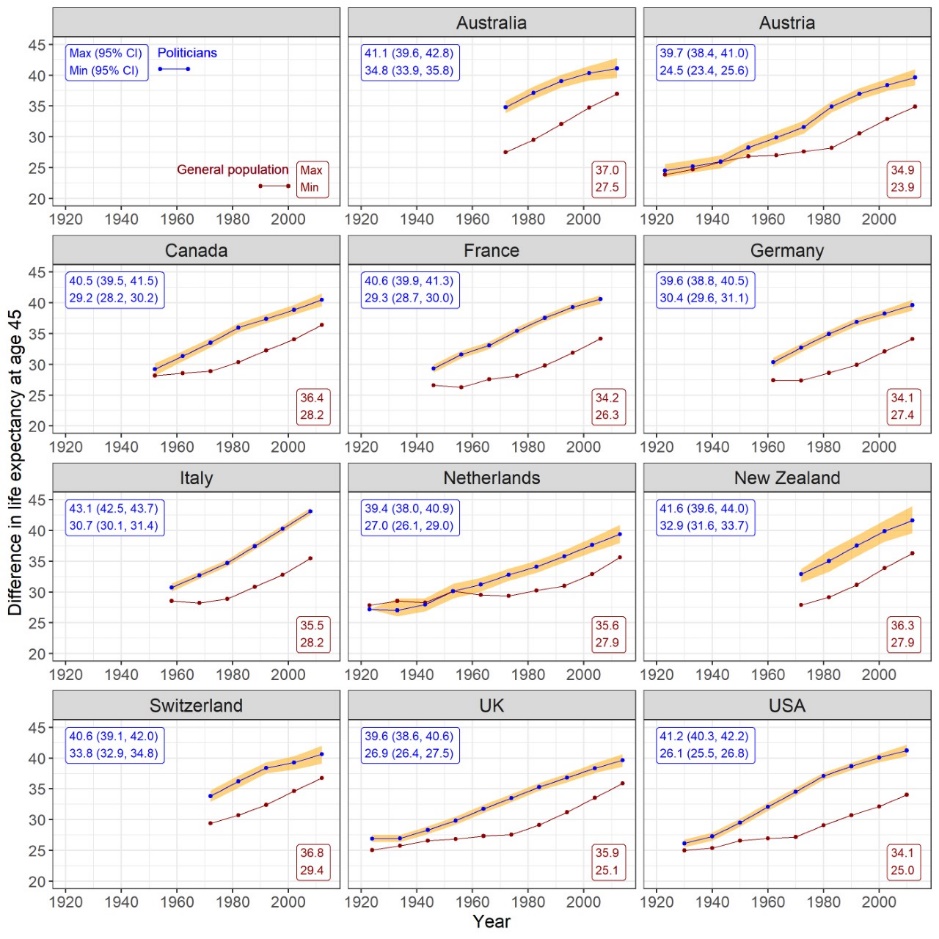


Shaded orange areas are the 95% CIs of the politicians’ life expectancies.

**S8. Subgroup analysis: Gaps in remaining life expectancies for males**

Fig. S8. Gaps in remaining life expectancies at age 45 between male politicians and general populations in 11 countries.


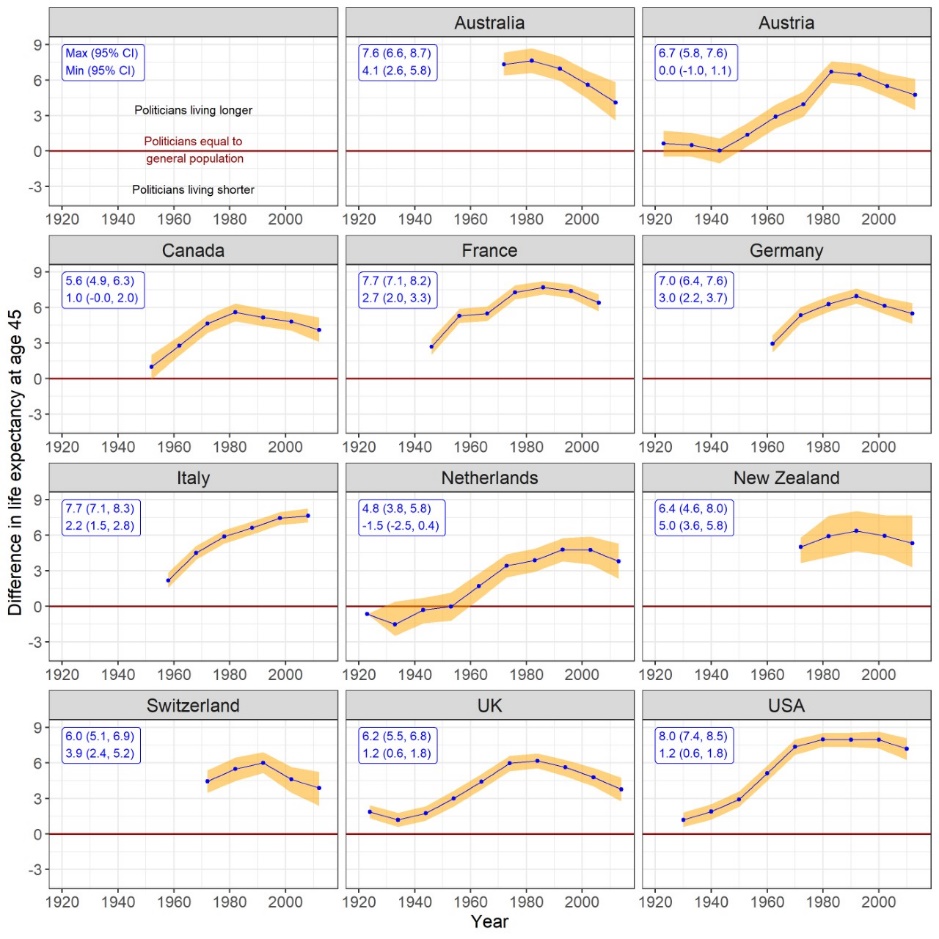


Shaded orange areas are the 95% CIs of the politicians’ life expectancies.

**S9. Subgroup analysis: Remaining life expectancies for females**

Fig. S9. Remaining life expectancies at age 45 for female politicians and general populations in 11 countries.


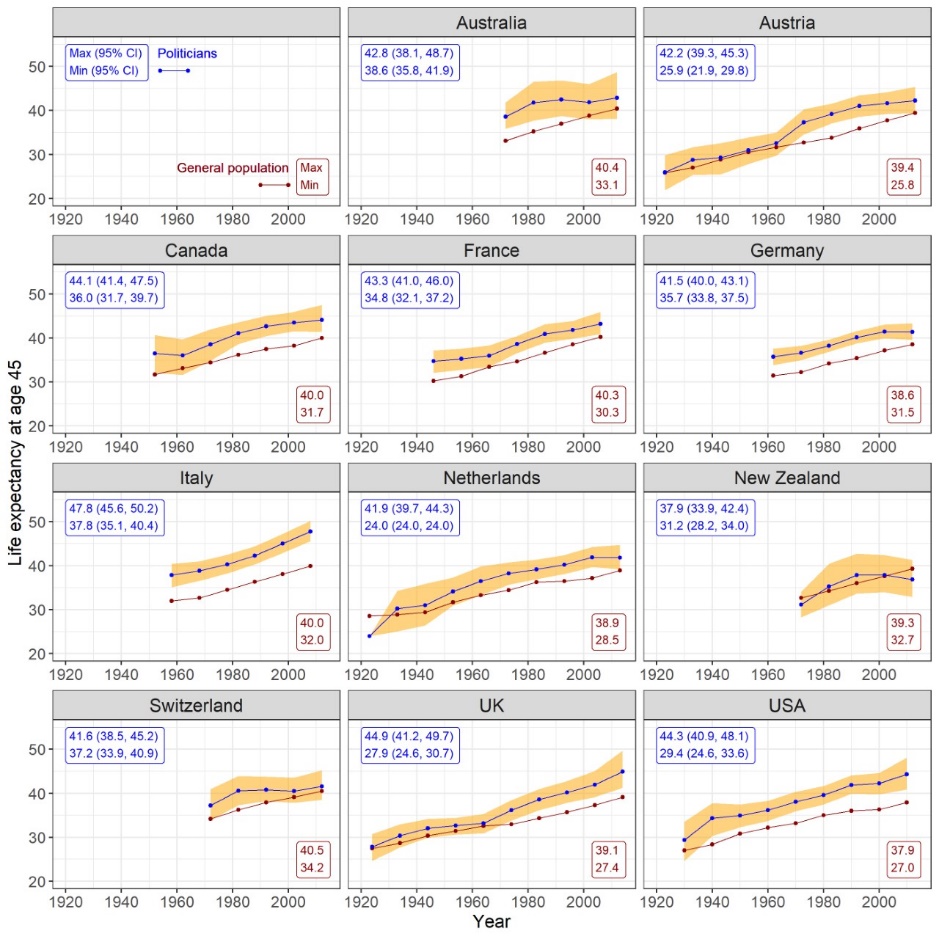


Shaded orange areas are the 95% CIs of the politicians’ life expectancies.

**S10. Subgroup analysis: Gaps in remaining life expectancies for females**

Fig. S10. Gaps in remaining life expectancies at age 45 between female politicians and general populations in 11 countries.

#
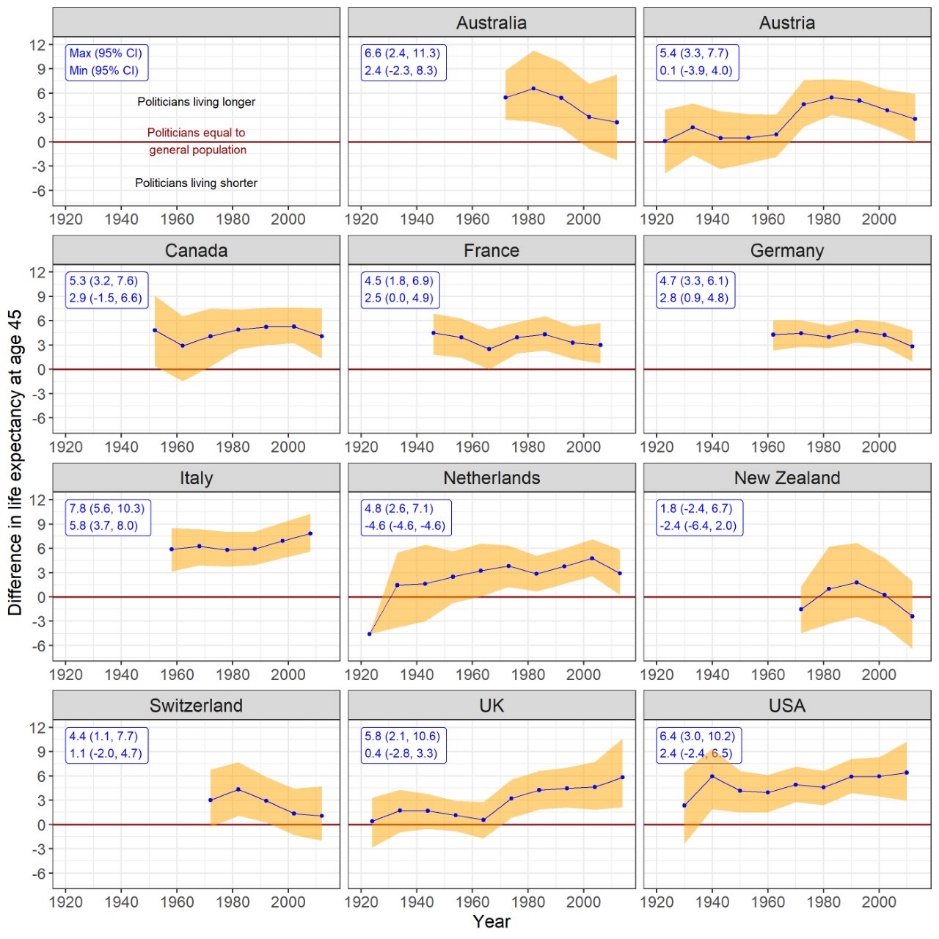


# Shaded orange areas are the 95% CIs.

**S11. Goodness of fit –** **Cumulative hazard plots based on Cox-Snell residuals**

Figure S11: Plots of cumulative hazard of Cox-Snell residuals against the Cox-Snell residuals for different periods in each country. A straight line with a unit slope and a zero intercept indicates a satisfactory model fit.

Australia


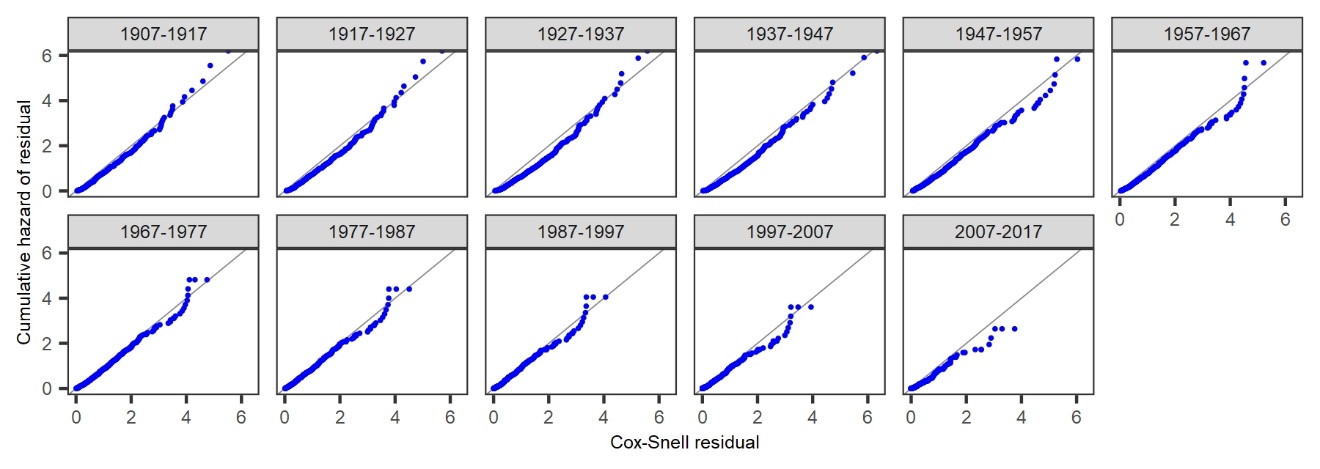


Austria


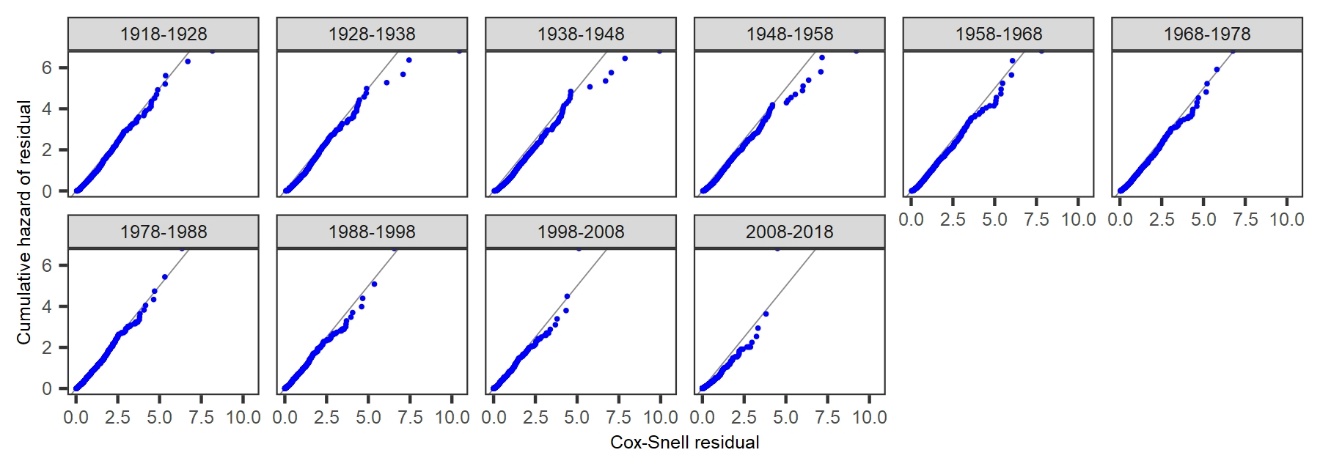


Canada


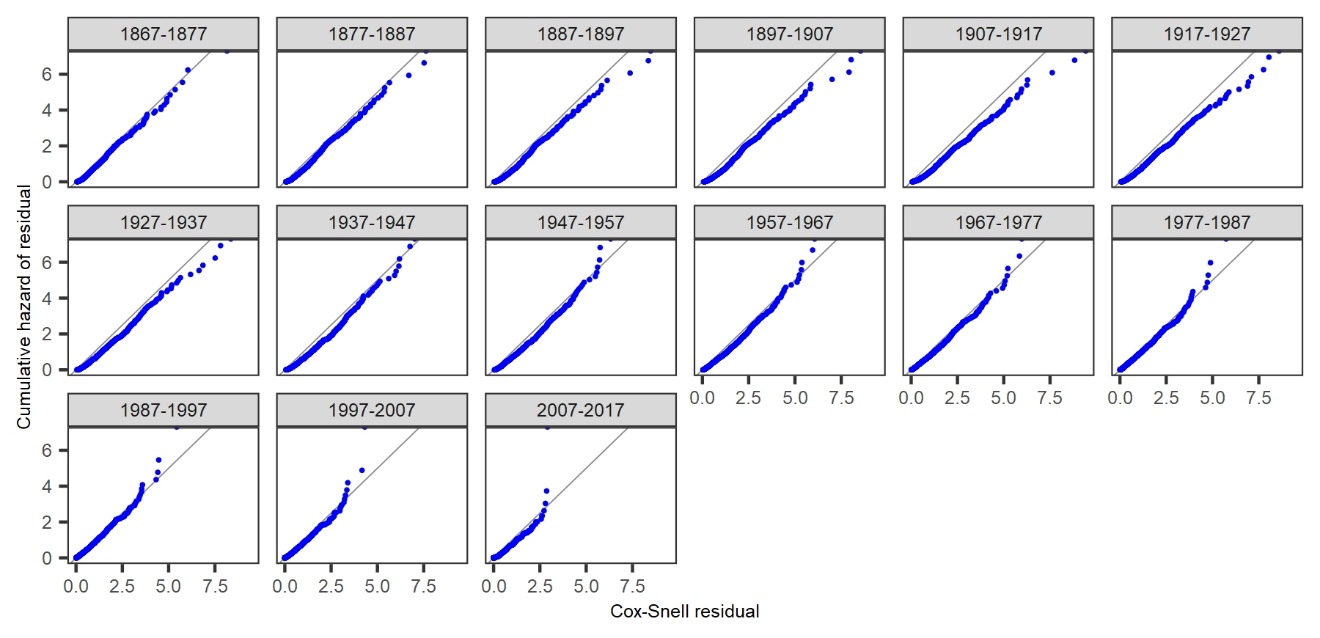


France


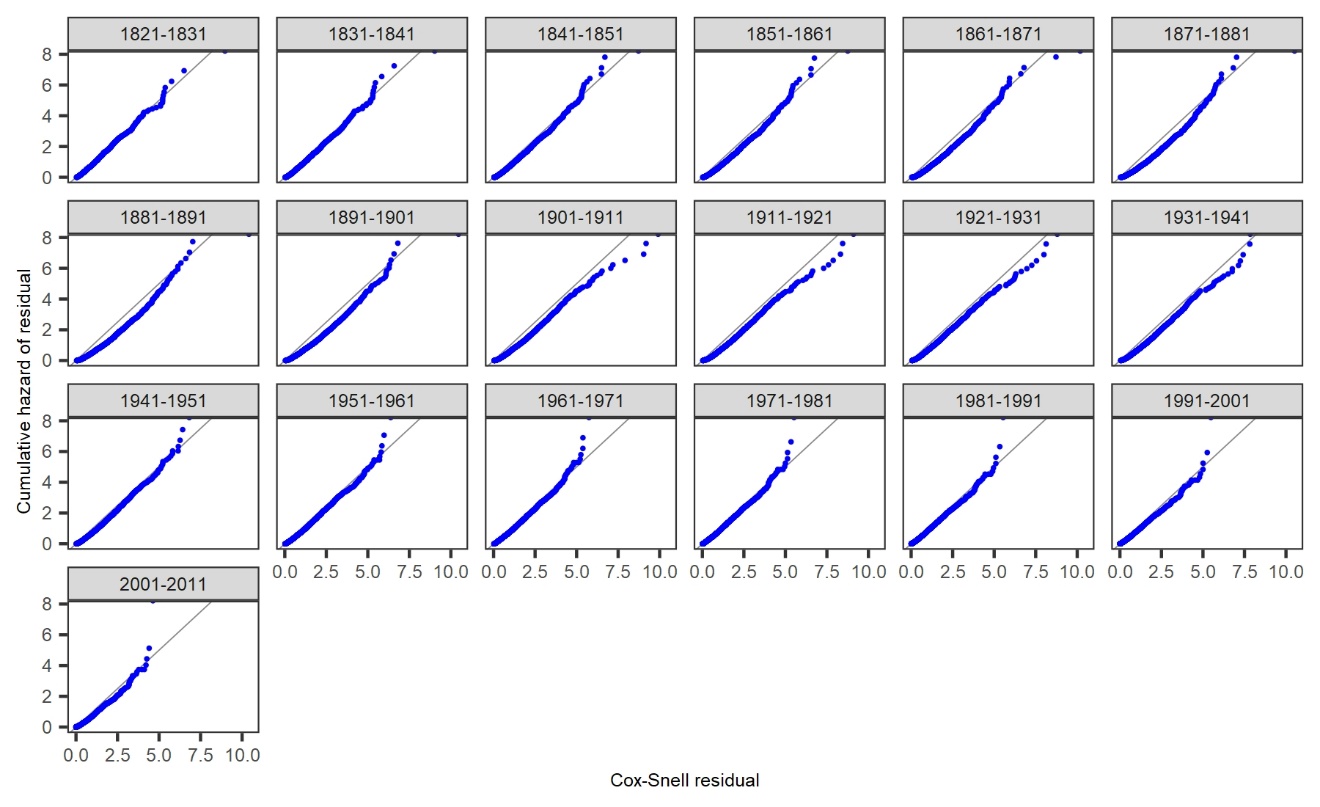


Germany


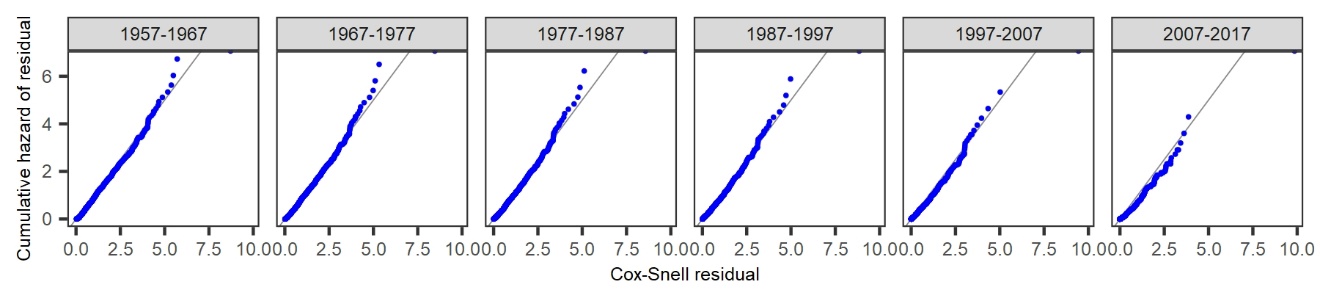


Italy


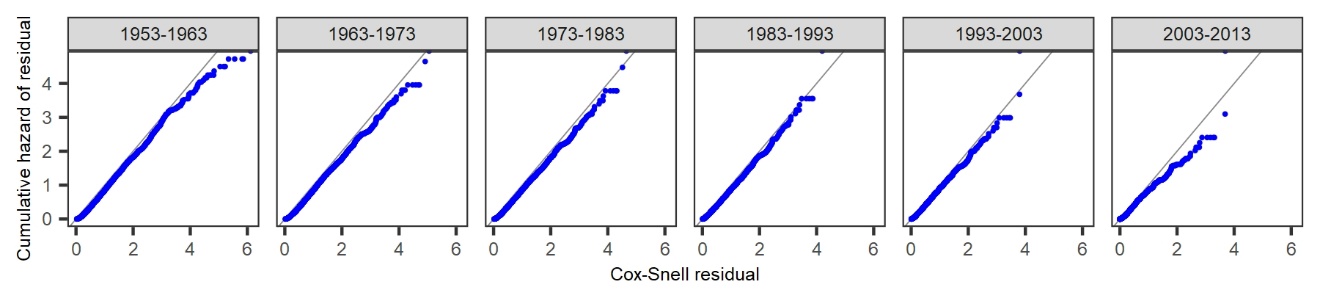


Netherlands


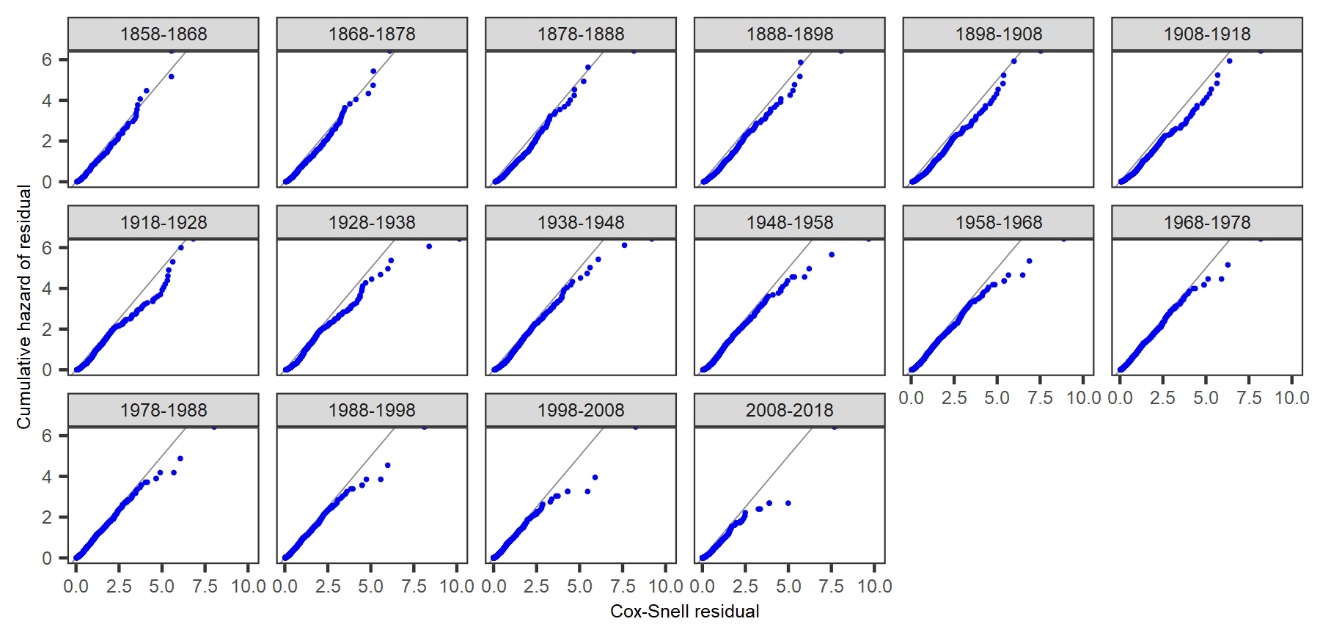


New Zealand


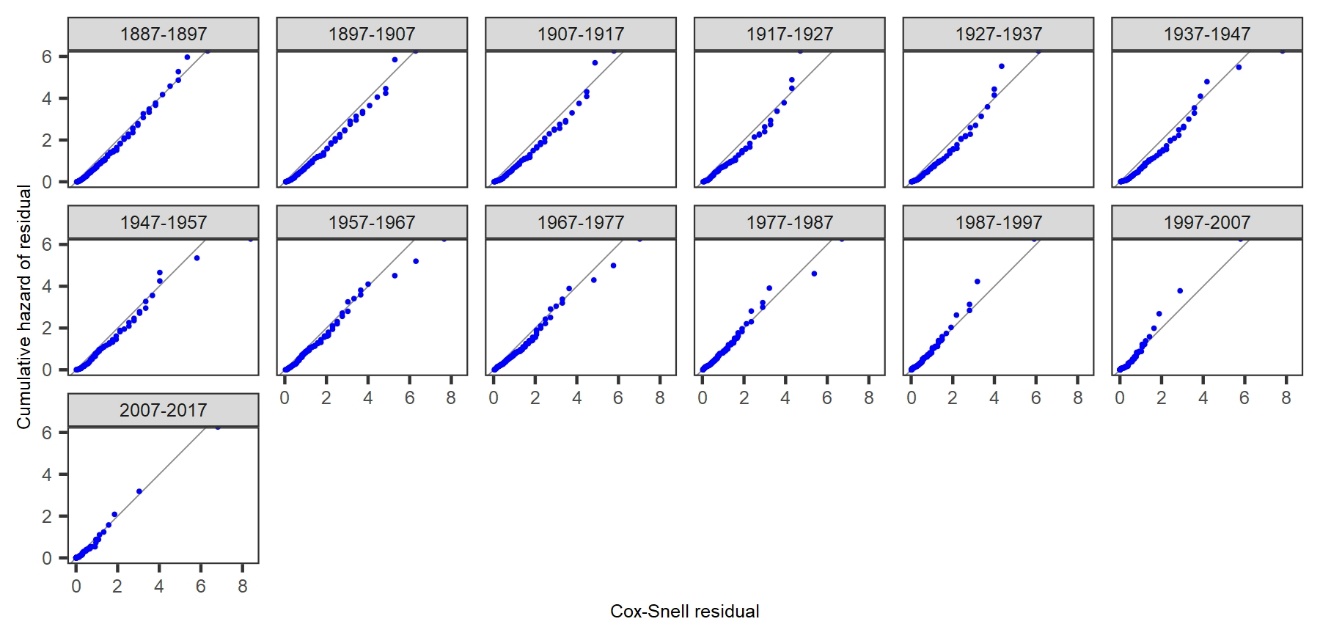


Switzerland
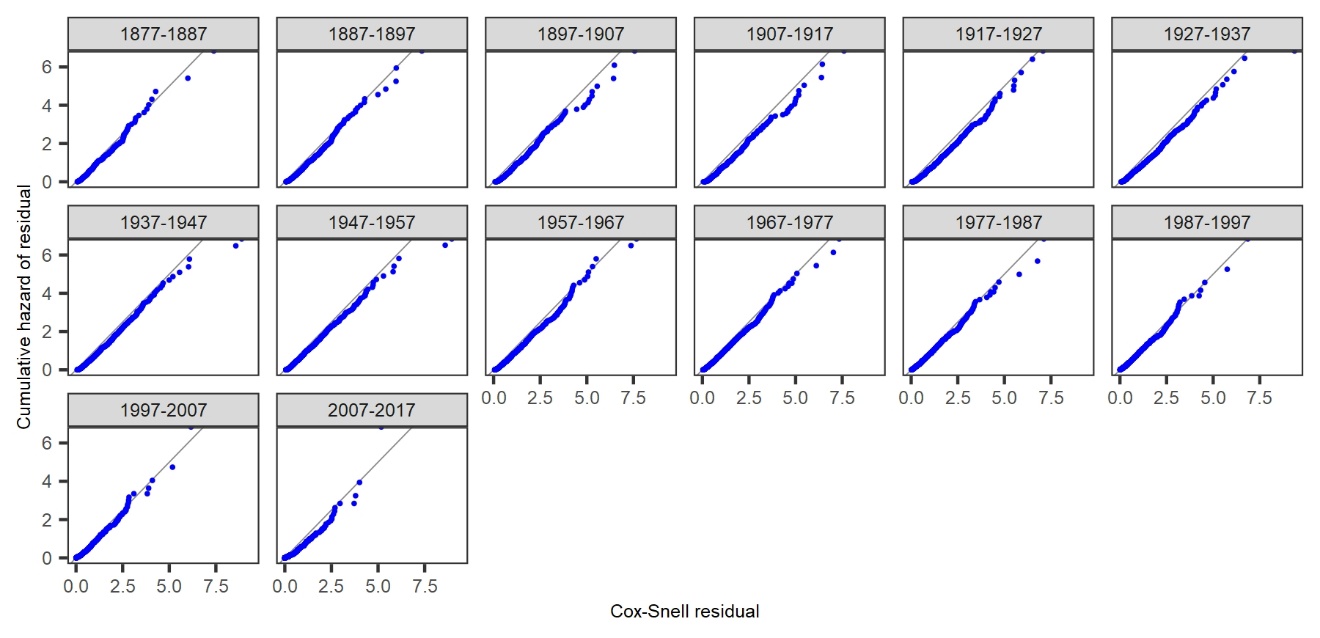


United Kingdom


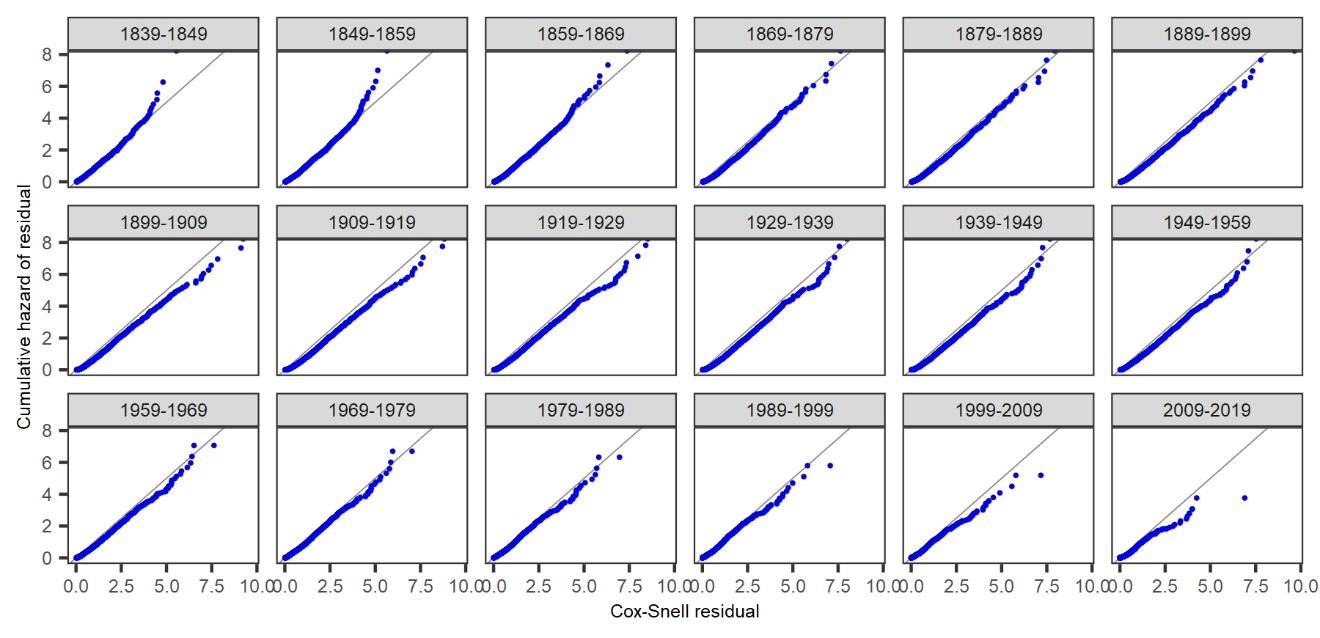


United States


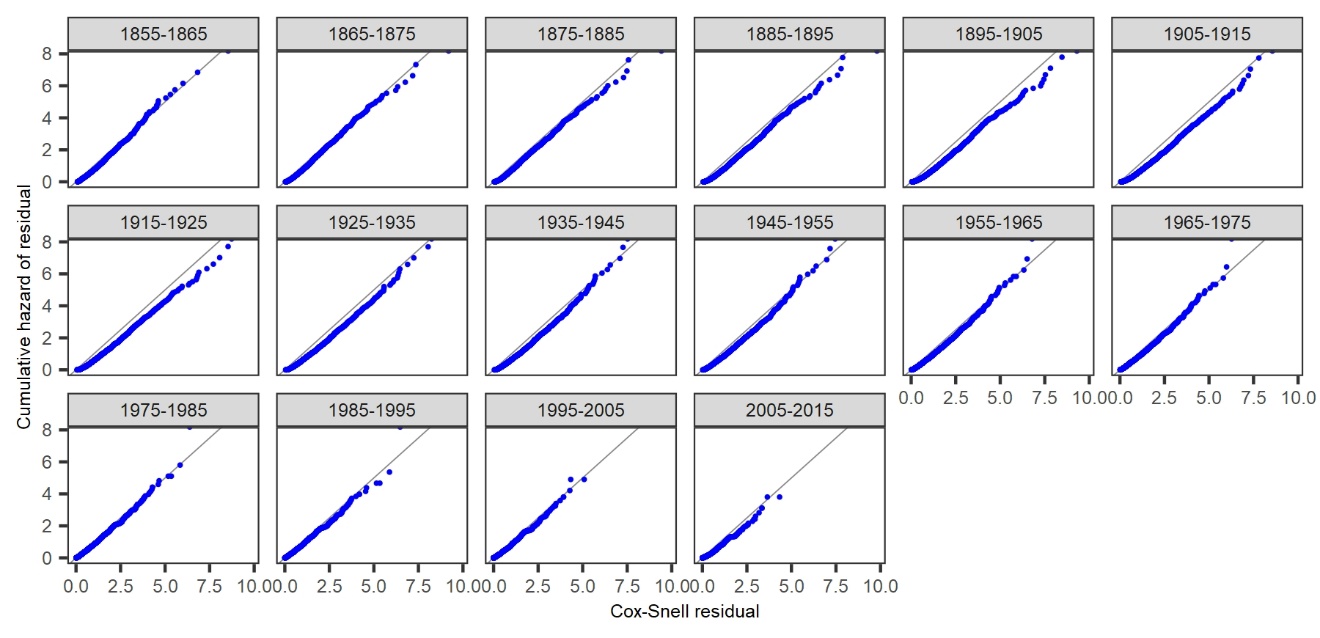


**S12. Goodness of fit –** **Agreement between observed and predicted survival rates**

Figure S12: The observed (Kaplan-Meier estimates – blue lines) and the predicted (Gompertz proportional hazards models - red lines) survival rates for different periods in each country

Australia


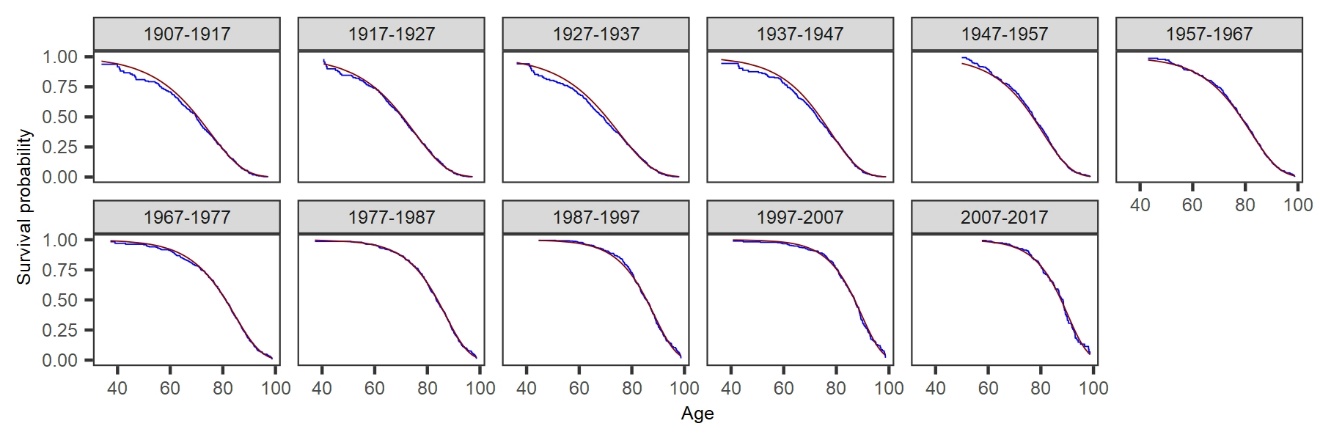


Austria


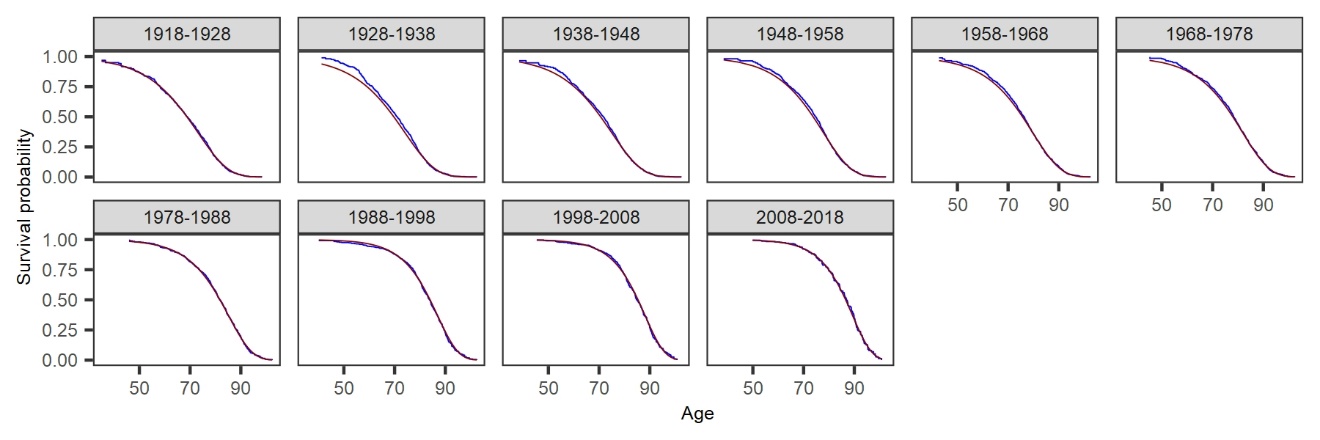


Canada


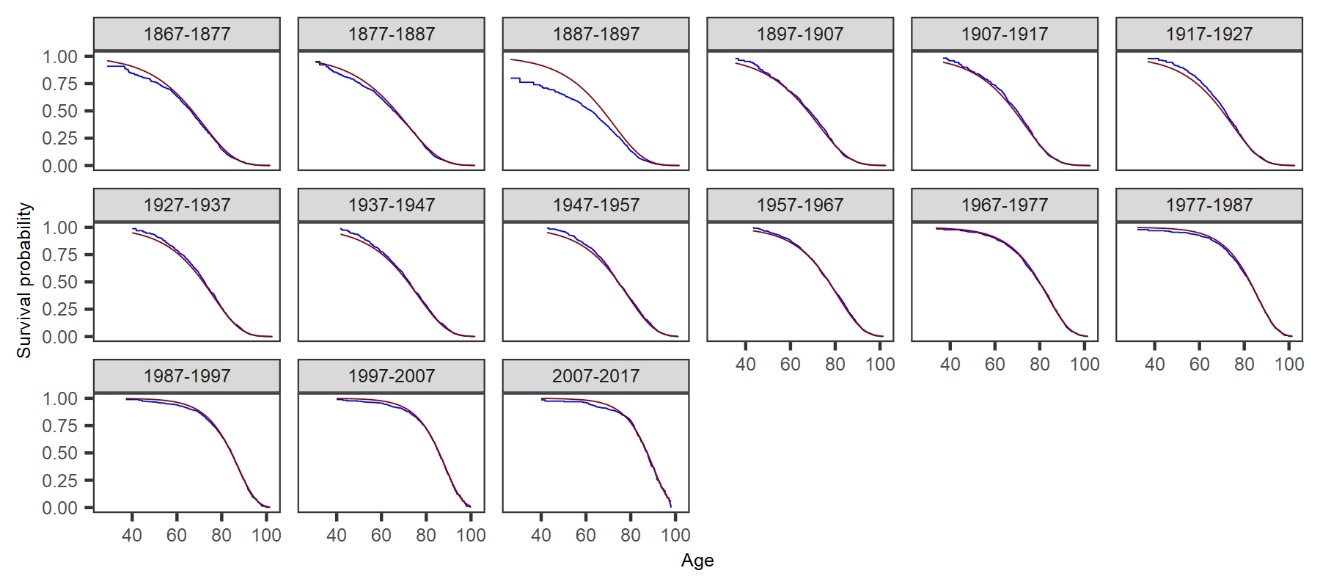


France
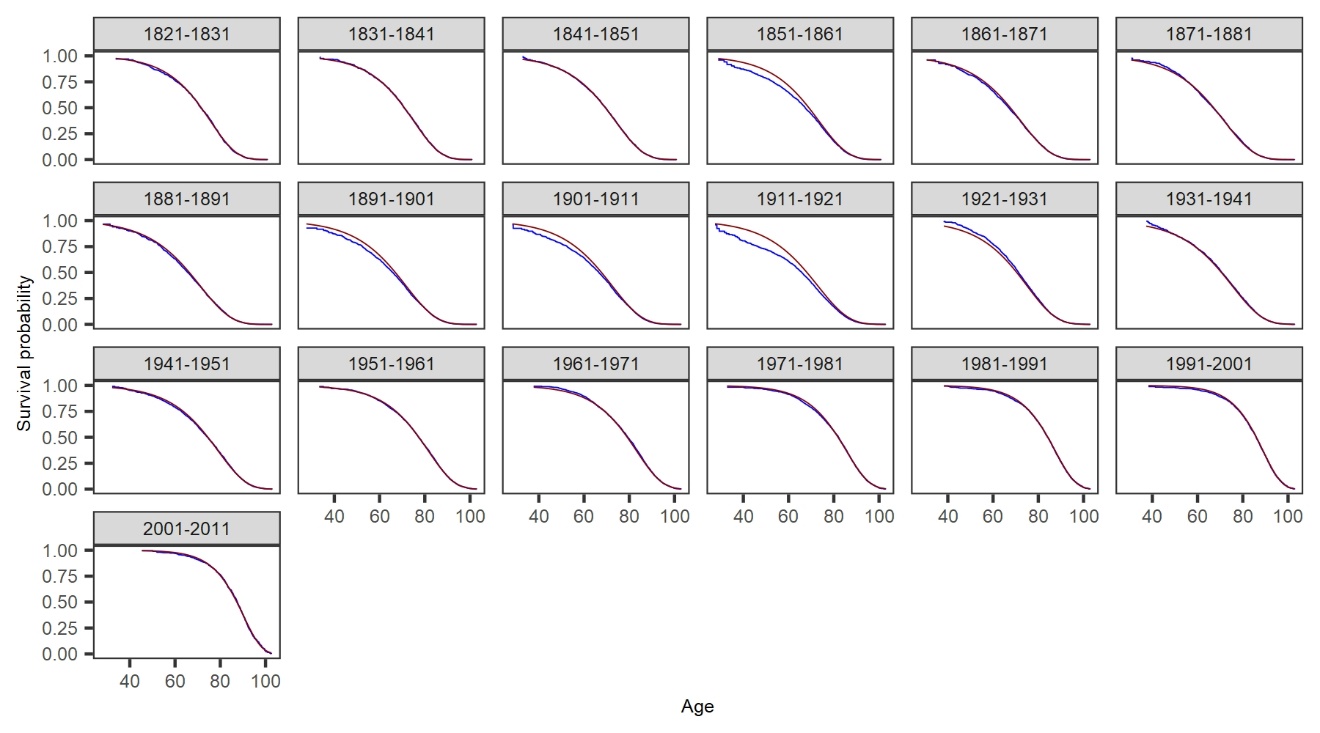


Germany


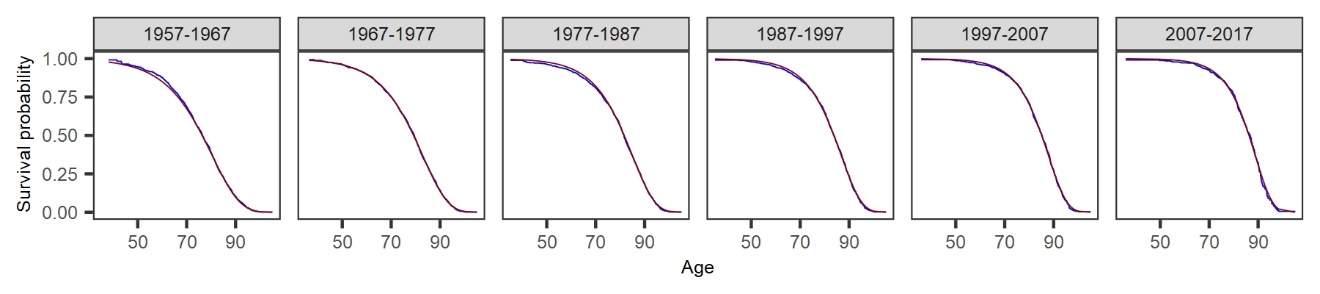


Italy


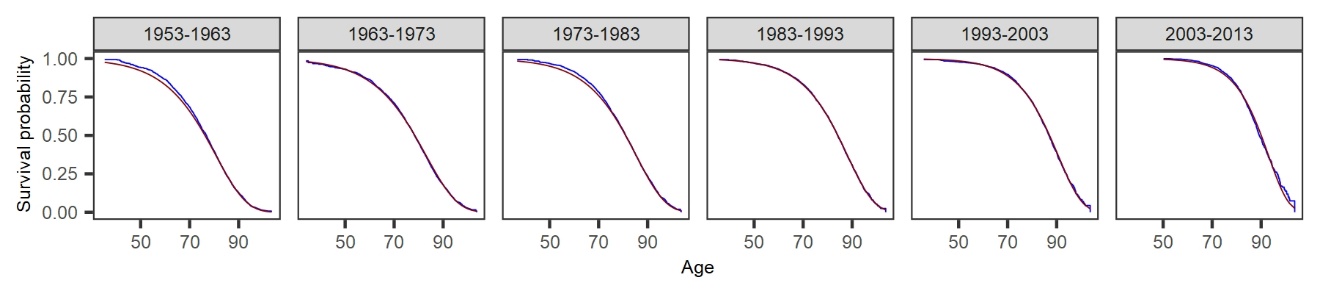


Netherlands
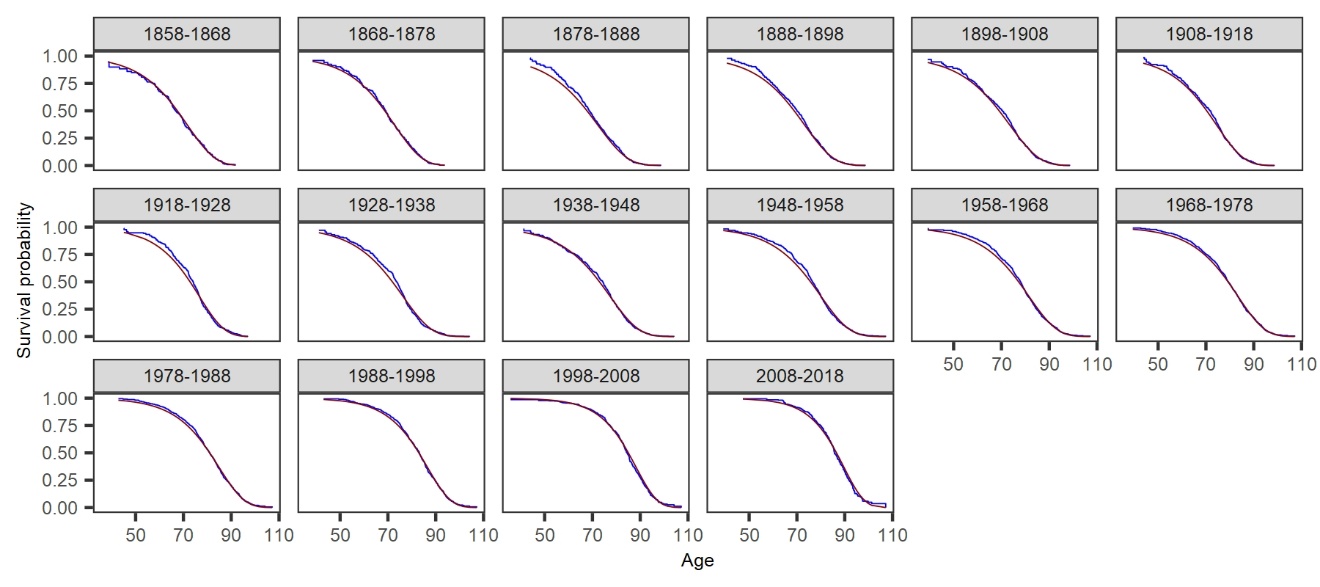


New Zealand


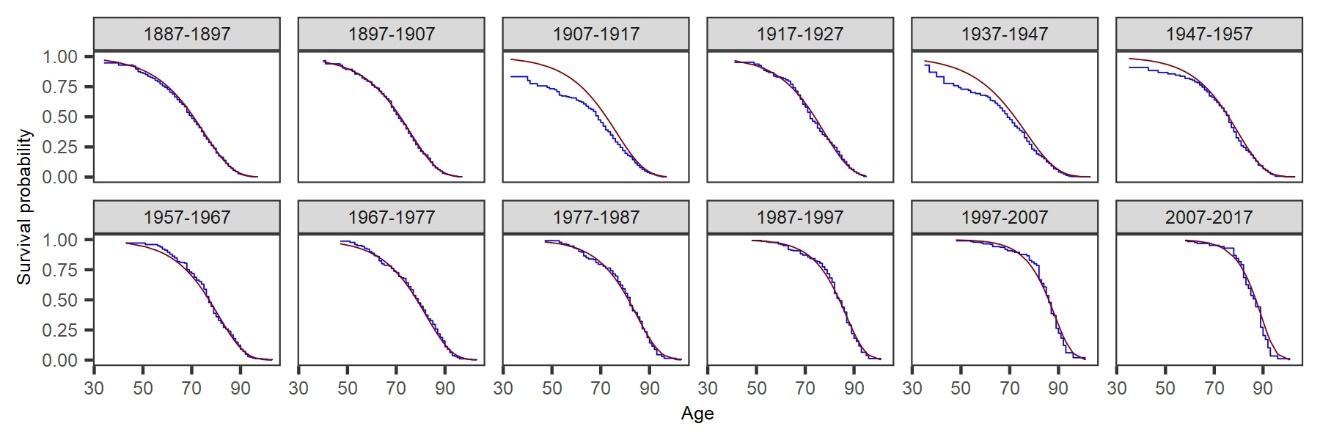


Switzerland
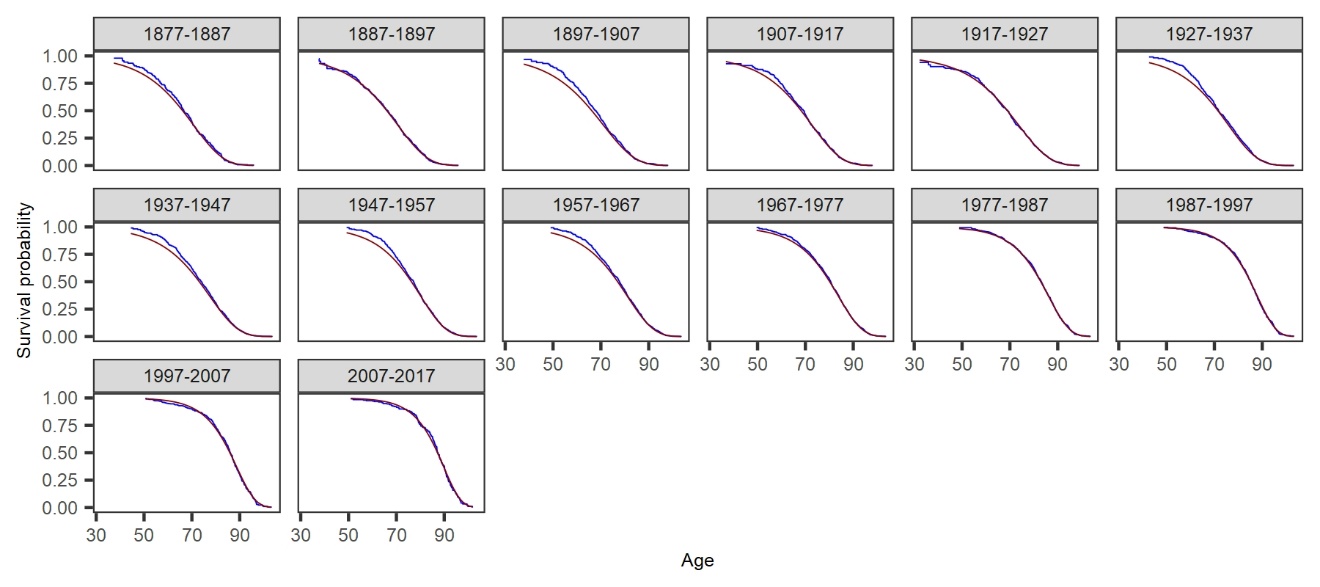


United Kingdom
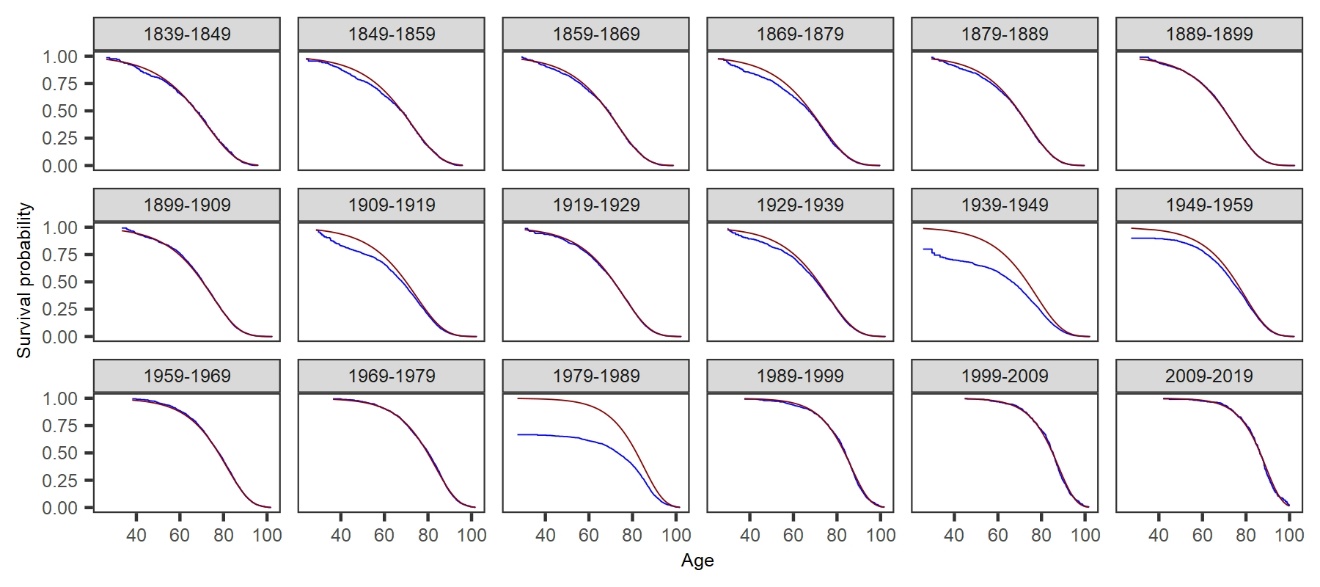


United States
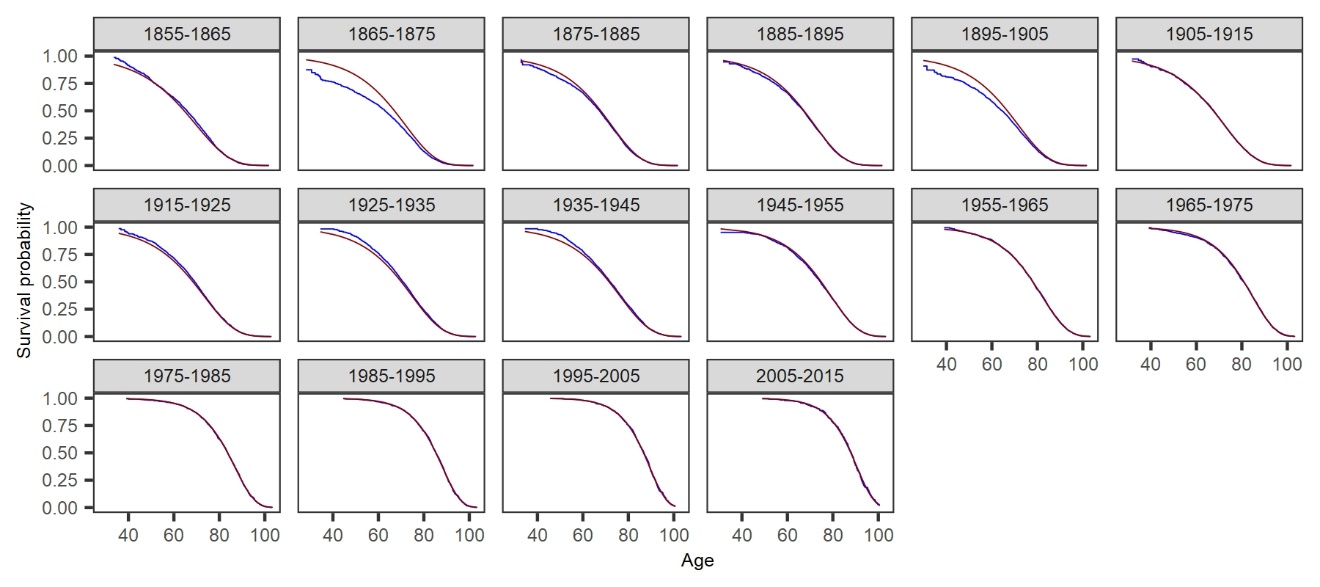

Supplement: Supplementary file 1 — Supplementary file1 (DOCX 5322 kb) [file 10654_2022_885_MOESM1_ESM.docx]
